# Ending the single versus multi-bedroom debate: Hybrid rooms meet stroke rehabilitation needs

**DOI:** 10.1371/journal.pone.0356623

**Published:** 2026-09-03

**Authors:** Marcus White, Mark Huen Choong Lam, Tianyi Yang, Ruby Lipson-Smith, Juan Pablo Saa, Aaron Davis, Mehrnoush Latifi Khorasgani, Julie Bernhardt, on behalf of NOVELL Redesign

**Affiliations:** 1 STRÜDAL, Centre for Design Innovation, Swinburne University of Technology, Hawthorn, Victoria, Australia; 2 Faculty of Architecture, Building and Planning, The University of Melbourne, Victoria, Australia; 3 The Florey Institute of Neuroscience and Mental Health, Heidelberg, Victoria, Australia; 4 The MARCS Institute for Brain, Behaviour and Development, Western Sydney University, Westmead, New South Wales, Australia; 5 La Trobe University, Bundoora, Victoria, Australia; 6 The University of South Australia, UniSA Creative, Adelaide, South Australia, Australia; The Hong Kong Polytechnic University, HONG KONG

## Abstract

Stroke is a leading cause of disability worldwide, and stroke rehabilitation plays a critical role in recovery. Early rehabilitation often occurs in a hospital setting, but the hospital built environment is rarely designed to support the specific needs and priorities of people who have had a stroke. In recent decades, there has been an ongoing debate regarding the benefits of single versus multi-bed rooms in healthcare settings. This debate centres around balancing privacy and rest, with social interaction and safety. While single rooms may be beneficial for some people, multi-bed rooms may be better for others. Both options, however, come with their own set of drawbacks in the context of stroke rehabilitation. In this study, we conducted a collaborative Living Lab to design a series of provocative-prototype hybrid room typologies that merged different aspects of single and multi-bed rooms to provide options specific for stroke rehabilitation. These typologies were used to generate four alternative bedroom designs which were modelled in virtual reality. The designs were informed by a values-based brief, outlining a set of predefined key criteria for optimising stroke rehabilitation environments: opportunities for activity and rest, wellbeing, safety, efficiency, flexibility, personal control, access to outdoors and green space, and access to a positive and stimulating environment. Twenty lived-experience and professional experts evaluated these virtual designs against the predefined values-based criteria. Evaluation revealed two preferred hybrid hospital room designs that incorporated beneficial features of both multi-bed and single-bed rooms. Our approach of values-based design and evaluation within a virtual environment proved an effective means of generating and evaluating design options with meaningful input from a wide range of stakeholders. These findings highlight the potential for flexible and adaptable room design to meet the specific needs and priorities of people participating in early-stage rehabilitation after stroke.

## Introduction

### Stroke, rehabilitation, and built rehabilitation environment

Stroke is a major cause of disability and mortality globally [1]. In 2023, the lifetime economic burden associated with stroke in Australia alone was estimated to exceed AUD 15.7 billion, equating to over AUD 350,000 per individual affected [2]. By 2050, annual stroke incidence in Australia is projected to reach 72,000, including 55,000 people with first-time stroke and 17,000 people with recurrent strokes [2].

Stroke rehabilitation is typically a long-term process that spans years. It starts in a hospital setting, gradually transitioning to outpatient care and then to community support. In Australia, after receiving acute care in hospital, over 50% of people with stroke require ongoing rehabilitation, which is typically initially provided in specialised inpatient rehabilitation facilities [3]. Targeted therapy and repetitive practice are central to rehabilitation, and people who have had a stroke are encouraged to be physically, cognitively, and socially active outside of their scheduled therapy time to further promote their recovery [4,5]. While recovery can take years, in Australia, the inpatient rehabilitation phase usually lasts from several weeks to months [6] and coincides with a critical stage for neurological recovery [5]. The care and rehabilitation experience received in inpatient rehabilitation facilities is therefore critical to recovery, wellbeing, quality of life, and has downstream societal and economic impacts [2].

The design and built environment of hospitals can impact care processes, wellbeing, and clinical outcomes [7,8]. The needs and priorities in subacute rehabilitation are distinct from other clinical contexts, with greater emphasis on encouraging autonomy for people with stroke, promoting activity and practice, rest for neurological repair, and consideration of falls risks [9]. There is an emerging evidence-base for the relationship between the healthcare built environment and stroke rehabilitation [10], with research emphasising the importance of architectural layout, access to nature, and flexible design to support activity, wellbeing, and safety [11].

Currently, the design of healthcare facilities is largely driven by strict guidelines and cost considerations [12], making it inflexible to adapt to the specific needs of stroke rehabilitation. Rehabilitation facilities are often designed similarly to acute hospital settings, despite the needs and priorities of subacute care being very different to acute care [13]. Many stroke survivors spend over 75% of their hospital time in their bedrooms [14], and bedroom design is critically important for promoting activity, rest, wellbeing, safety, and efficient care for optimal rehabilitation [14,15]. There is therefore a need to develop new, innovative designs for hospital bedrooms that address the specific needs of stroke rehabilitation care.

### Stakeholder-informed design principles for stroke rehabilitation bedroom environments

The enduring debate regarding the relative merits of single versus multi-bed room configurations remains unresolved in both academic literature and clinical practice [10,16–18]. Existing evidence suggests single rooms offer distinct advantages in terms of privacy and environmental control, while multi-bed configurations, demonstrate benefits in social connectivity, clinical efficiency and safety outcomes [9,19]. A review of research concerning hospital room design for people with neurological conditions concluded that single rooms offer advantages in patient privacy, and control over light and sound providing better quality rest, while multi-bed rooms were associated with slightly reduced fall rates, shorter lengths of stay, improved quality of care, enhanced social connectivity, and better mood among patients [19]. Additionally, in a review of studies examining various factors that influence the design of rooms for people who have had a stroke, Daemen [20] identified key features that contribute to good room design. These included views of nature, availability of daylight, along with sound, architectural elements, the inclusion of family zones within patient rooms, indoor plants, and flooring materials. The impact of these factors was assessed in relation to hospital-acquired infections, sleep quality, patient privacy, social interaction, overall patient satisfaction, the healing process, levels of stress and anxiety, pain, appetite, and the incidence of falls [20]. While each room type provides clear benefits, with single rooms providing better privacy and environment control for quite rest, and multi-bed rooms providing better environments for social connectivity, and more efficiency and safety, there is no definitive evidence favouring one over the other [9,19].

### The present study

Recognising the importance and complexity of designing for stroke rehabilitation, we established the Neuroscience Optimised Virtual Environment Living Lab (NOVELL) as a collaborative platform for innovation of inpatient stroke rehabilitation environments. This initiative brought together over 130 stakeholders, including people with stroke, clinicians, policy makers, and design professionals, through an iterative co-design process.

In early phases of NOVELL, we employed value-focused thinking methodologies [21] to systematically identify stakeholder priorities in stroke rehabilitation facility design [22,23]. Through this process, we established four ‘fundamental objectives’, core values that define what is important in the built environment to optimise stroke rehabilitation. Namely, rehabilitation facilities should: 1) maximise opportunities for activity (physical, cognitive, and social) and rest for people with stroke; 2) maximise wellbeing for people with stroke, staff, and visitors; 3) maximise safety for people with stroke, staff, and visitors; and 4) maximise the efficiency of care, including reducing time and cost [22,23]. We also identified nineteen ‘means objectives’, design requirements that articulate optimal conditions for rehabilitation environments. These included critical considerations such as maximising personal control to enhance privacy and wellbeing, and creating positive, stimulating spaces to facilitate therapeutic activity [22,23].

The present study reports later phases of the NOVELL project. The aim of the present study was to use our pre-defined values-based objectives to design and evaluate new hybrid solutions for bedrooms for stroke rehabilitation facilities that synthesise the perceived benefits of single and multi-bed rooms. We ask the research questions: what are the key elements of single and multi-bed rooms that are beneficial to rehabilitation? Can we gain a better understanding of these elements though collaborative stakeholder engagement? And finally, can these beneficial elements of both single and multi-bed rooms be achieved through co-creation of new designs of ‘hybrid’ room typologies?

## Method

**Fig 1:**
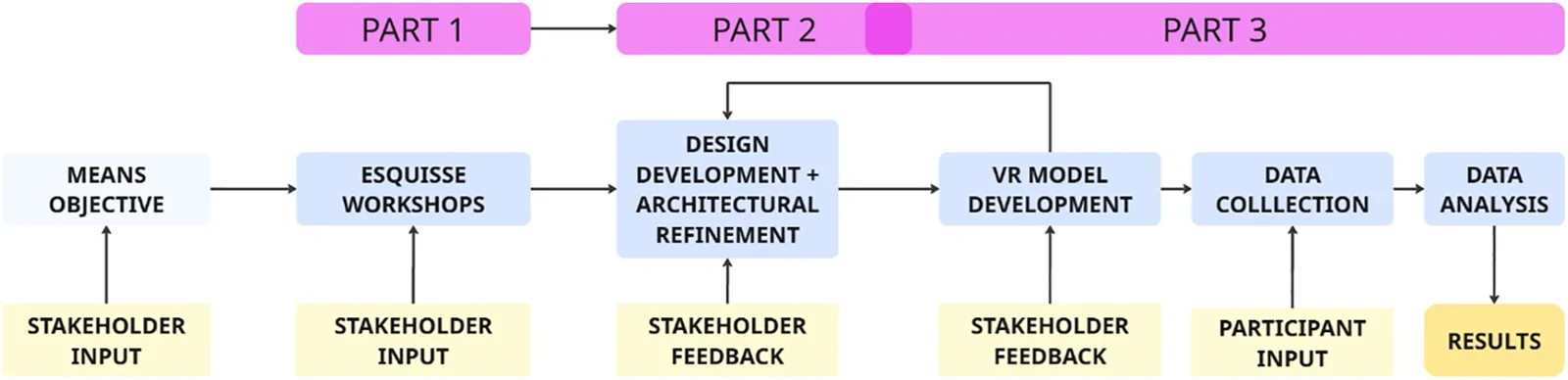
Methodology flowchart. Flowchart summarising the methodology used to determine the ideal hospital room type for stroke rehabilitation.

To address the aim of this study, we broke down our methodology into three parts [Fig 1]. Firstly, we developed speculative design briefs and targeted design-research questions based on the values and objectives defined in previous multi-stakeholder workshops fundamental and means objectives that informed our bedroom design briefs are detailed elsewhere [22,23]. We incorporated these briefs and questions into a series of *‘Provotype Esquissé Workshops’,* where we used rapid design sketching processes to generate provocative, speculative design prototypes (Fig 1). Secondly, we commenced a *design development and architectural refinement* process for the room designs, testing them through a variety of digital 3D modelling techniques, and continuing stakeholder feedback (Fig 1). Finally, we created *virtual reality (VR) testing models* of the final bedroom designs and asked stakeholders to view and evaluate these (Fig 1). We then analysed resulting data to determine if the designs met the predefined objectives for optimal stroke rehabilitation environments.

### Part 1: Speculative design - ‘Provotype Esquissé Workshops’

To promote experimentation, explore multiple concepts quickly, and encourage speculative ‘blue sky’ creative thinking, we developed a workshop approach that drew from the ‘speculative design’ methodology described by Dunne & Raby [24], combined with the exaggerated ‘up to 11’ approach of White [25], and a provocation-prototyping approach described by Boer & Donovan [26]. We developed a system of rapid digital sketching drawing from the traditional method called *Esquissé* [27], a process, derived from the Italian word *schizzò*, which involved rapid, iterative hand sketching of ideas, in a workshop format inspired by the ‘design charrette’ [28]. A design charrette is a collaborative design workshop (popular in urban design spheres since the 1970s, and more recently appropriated as the ‘design sprint’ in tech innovation groups), that involves designers, stakeholders and end users working on a specific design project to ideate and explore possibilities [29]. We combined digital sketching tools (such as android and ipad tablets, and windows devices with stylus capability), with mobile phone devices to directly record sketches, notes, diagrams, as well as copy-pasted images of architectural precedents, or other forms of inspiration, all combined using an online collaboration platform (Miro) and displayed on large format monitors in a university digital collaboration room, the iHUB, [30]. Workshops included both in-person and online participants, maximising opportunities for participation of diverse stakeholders, with the shared online space (Miro) functioning as a platform for dialogue and collaboration.

We ran six Provotype Esquissé Workshops with participant groups consisting of stroke survivors, clinicians, architects, designers, policymakers, and researchers. Within the workshops we set ten research questions and design provocations subset. These provocations were derived from key questions raised in the detailed briefing studies, to explore ways to improve stroke rehabilitation facilities. Participants were encouraged to experiment with the design issues and responses to create provocative design proposals that addressed the specific research question design provocations, and instructed to not concern themself with many of the other practical considerations that might normally limit such proposals, such as cost of construction. These proposals became design test case provocation prototypes, or ‘provotypes’ [26,31], and formed the basis for the next stage of design iteration, development and design testing.

### Part 2: Design development and architectural refinement

Over a six-month period, concepts from the Esquissé workshops, along with existing Australasian Health Facility Guidelines (AusHFG) and research on current built examples, were examined in detail in design workshops. In these workshops, various options were drawn, modelled in 3D and tested for accessibility, visual qualities, visual connection and view-quality using digital spatial analysis methods [32,33]. The focus was on creating overall room configurations, and the detailing individual components, such as balconies, window views, ease of staff supervision, connections to neighbouring areas and the ward, as well as ceiling types, materials, user-controlled features and furnishings, facilities to support family visit, automation and smart assistance, e-information boards, and colour choices. In-person and online weekly design reviews were held throughout this time to appraise and select options that were suitable. Designs were again shared with stakeholders on the Miro platform online and in person.

**Fig 2:**
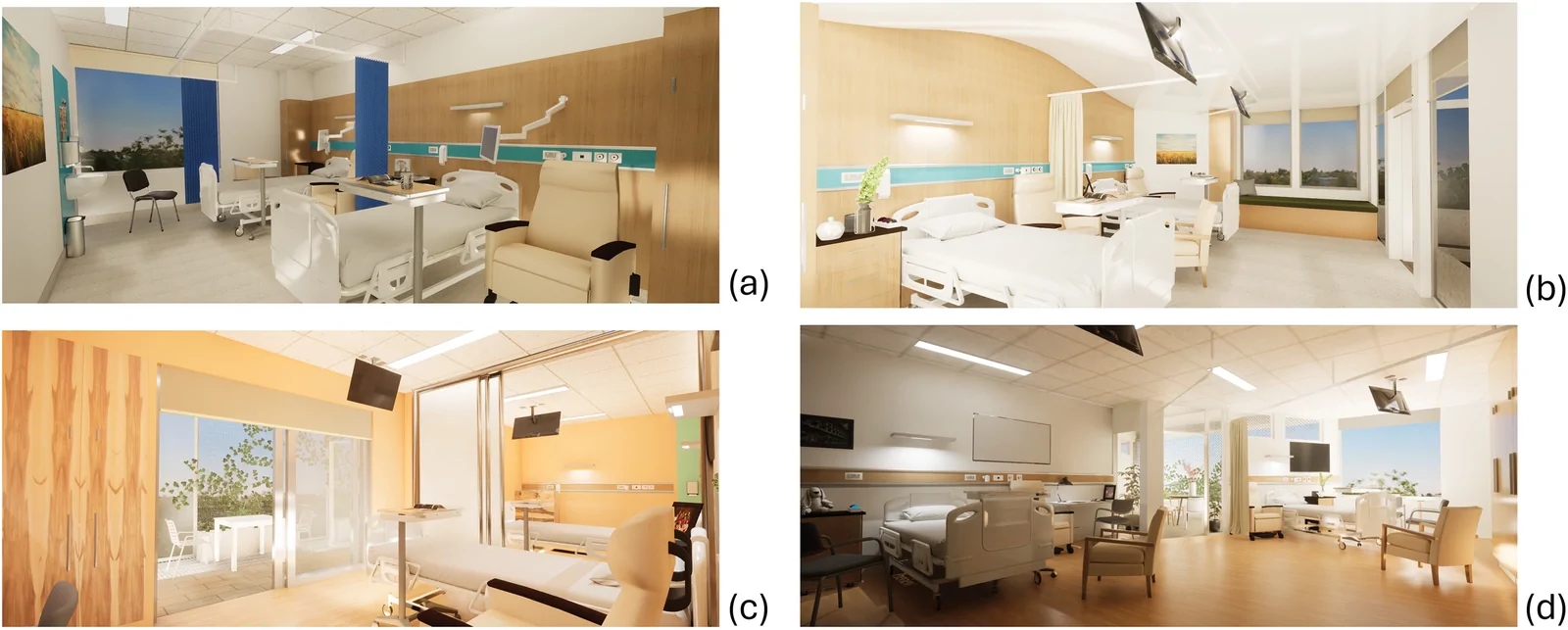
Screengrabs of four ‘Provotype’ stroke rehabilitation room. Each design emphasised on different means objective identified in our Detailed Brief. (a) Duet, (b) Troika, (c) Butterfly, (d) Quartet.

Four versions of hospital rehabilitation rooms were generated: Three new hybrid room designs, and one ‘baseline’ comparison design modelled on a ‘typical’ two-bed bedroom arrangement that are found in many current rehabilitation facilities [Fig 2] according to Yang et al.’s 2024 study [13]. Each room typology is detailed in the Results section of this paper.

### Part 3: Evaluating the bedrooms in virtual reality

We have previously developed a reproducible digital VR environments modelling approach which includes measures of user perception, as outlined by White et al. [34] and Yang et al. [35]. This VR approach has been successfully piloted with stroke survivors by Shannon et al. [36]. A variation of these approaches was further refined and developed for this study.

**Fig 3:**
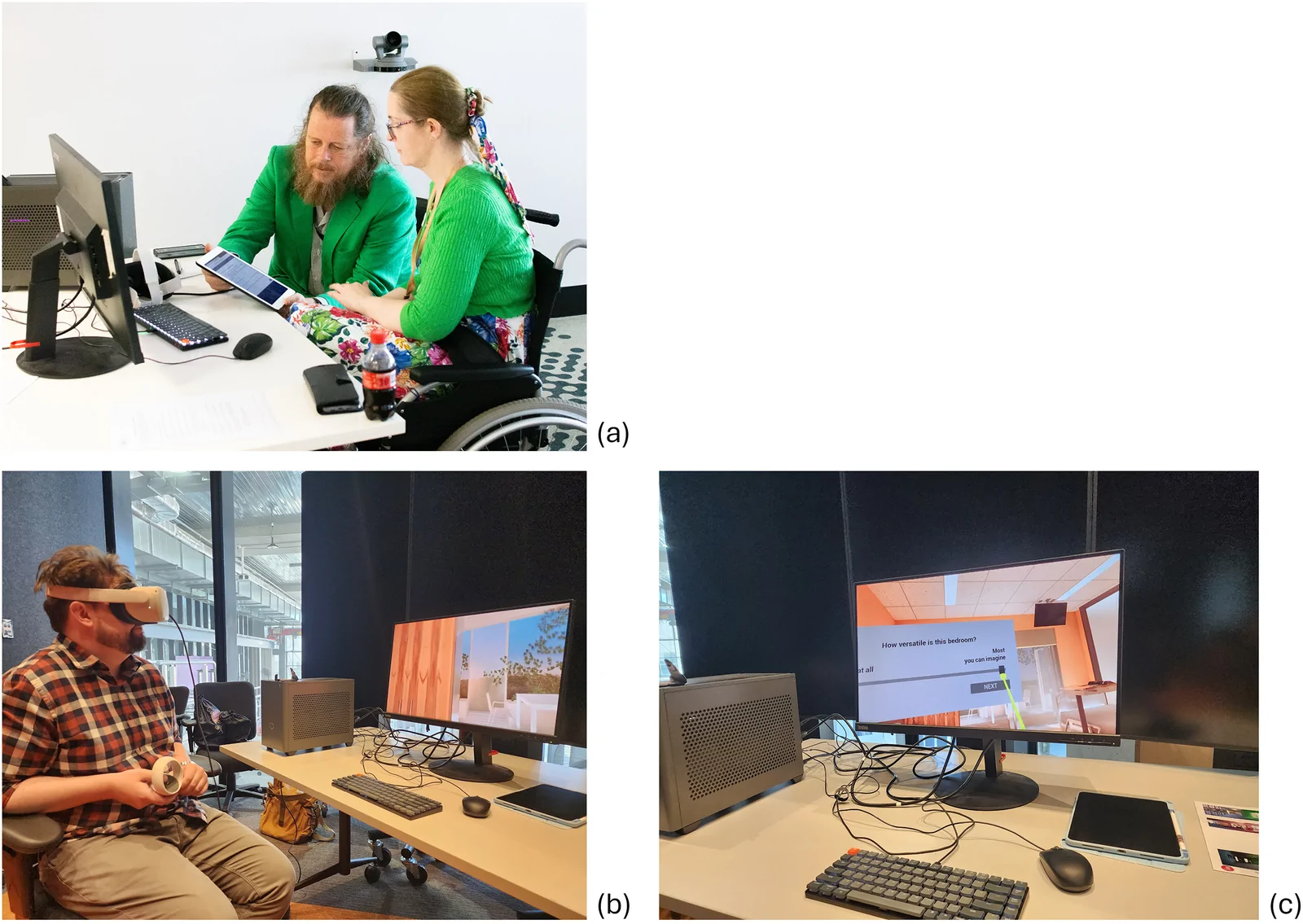
Photos showing participants taken for ‘tours’ of immersive virtual rehabilitation bedroom environments. (a) Prior to entering the VR environment, participants were asked to complete an online survey to obtain informed consent and demographic information. (b) Participants visited each bedroom within a VR environment. Audio descriptions of the bedrooms together with hospital background noise helped to explain what they are seeing and provide a more ‘realistic’ feel of the space. (c) Following each bedroom tour, a series of questions were asked about the room within the VR environment, where participants responded on a sliding Visual Analogue Scale (VAS).

The three provotype bedroom designs and the baseline comparison were integrated into a flexible, cross-platform building information modelling (BIM) management system that enabled efficient design modifications and comprehensive scenario testing [Fig 3]. The designs were then combined in a game engine where a VR testing environment was developed to capture participant preferences and emotional responses through interactive feedback options, Visual analogue scales were included within the VR environment for participants to provide feedback which has been shown to be reliable and internally consistent method based on exploratory studies by Shanen et al. [36], and Yang et al. [35] in the stroke rehabilitation contexts [37]. The VR sessions were complimented by semi-structured interviews to gain further insights into the participant’s opinions on each design.

### Recruitment of participants for VR evaluation

Target participants included people with expertise in one or more of the following disciplines: stroke rehabilitation clinicians; stroke rehabilitation academics; healthcare environments academics; academic researchers, architects, designers, and wayfinding consultants with experience of designing healthcare environments; healthcare service or healthcare design policy makers; and patient consumers with lived experience of stroke rehabilitation. Eligibility criteria included: 1) expertise in one of these target disciplines; 2) able to communicate in English (written and verbal); 3) 18 years of age or over; 4) physically able to sit upright and wear a VR headset and over-ear headphones; and 4) able to travel to central Melbourne, Australia. People with uncorrected vision or hearing loss or impairments were unable to participate. People with arm mobility impairments who could not operate the handheld controller were able to participate by giving verbal instruction to an assistant who operated the controller on their behalf. People who had previously experienced motion sickness from using VR in the past were ineligible to participate. People with stroke who had a significant language or communication impairment were invited to participated in an adapted VR evaluation process, the results of which will be published separately.

Experts were identified purposively through existing collaborative networks known to the researchers. Preference was given to people with expertise in two or more of the target disciplines so that we could get more informed views on the design options. Stroke survivors were offered a small honorarium for their time and to cover travel costs.

Twenty-one participants were recruited between 21 August 2023 and 23 October 2023. The sample size was not determined a priori, as no statistical comparisons were planned. The study prioritised depth over breadth; combining qualitative components with quantitative measures to explore whether VR-based preference elicitation could generate observable differences in preference between design options. Design evaluation using VR is a relatively novel method, particularly in a stroke survivor population, so we considered this study a proof-of-concept. Our final sample size was therefore kept relatively small, with an emphasis on achieving representation across each of the target expert disciplines.

Informed consent was verbal and responses were captured in a spreadsheet managed by the researchers. Participants were assigned a number, and data were anonymised and held in secure storage in accordance with Swinburne University Technology Privacy Procedures, Privacy Guidelines, and Research Data Management Policy, complying with the Privacy and Data Protection Act 2014 (Vic) and the Australian Code for the Responsible Conduct of Research [38].

### Scenario introduction and instructions

After providing informed consent, participants were fitted with their VR headset and headphones and provided with a simple explanation of the activity and guidance on how to use the hand-held controller. Participants then completed a sample ‘practice’ scene to familiarise themselves with the operation of the VR system and how to answer questions within the VR environment using the controller.

### Quantitative and qualitative evaluation of the four bedroom designs

After the participants had moved past the instructions, they viewed each of the four bedrooms (three new designs and one baseline control) in a random sequence. A sonorous audio voiceover described the bedrooms to the participants, explaining key features. Participants viewed each bedroom for between five and eight minutes while listening to the audio voiceover. Participants viewed the bedrooms from a seated (wheelchair) point of view, beginning just inside the door to the room but could move within a limited area and rotate 360 degrees within the room. Key features within the room such as blinds, curtains, sliding doors and moving ceilings were animated to describe how they work.

Participants were encouraged to speak their thoughts whilst in the VR, as part of a ‘think aloud protocol’ [39] and their responses were audio-recorded. Once the audio voiceover had completed and participants indicated that they were finished viewing the room, participants were asked to give a verbal summary of their first impressions of the room before removing their headset.

With their headset still on, participants were then asked to answer a series of five evaluation questions to assess how well the room met the predefined core objectives that were used to generate the designs. The evaluation questions were presented as a series of ‘Visual Analogue Scales’ (VAS) [34,36,40,41] displayed within the VR room environment [Fig 3c]. To answer these VAS questions, participants used their hand-held controller to move a slider on a semi-transparent block, adjusting the slider anywhere along the scale between 0 and 100 to indicate their preferences [Fig 3c]. Asking participants to complete VASs within the VR environment has been shown to be a reliable and internally consistent method based on exploratory studies by Shannon et al. [36], and Yang et al. [35] in stroke rehabilitation contexts. The five VAS evaluation questions and their endpoint anchors are detailed in Table 1.

**Table 1:** The key objectives that informed the design brief, and the corresponding Visual Analogue Scale (VAS) questions that were displayed within the VR environment to assess the extent to which these objectives had been met.

Key objectives	VAS questions displayed to participants in VR environment		
	Preamble	Question	Endpoint anchors
Instrumentally important:			
Versatility.	Think about the versatility and flexibility of this bedroom. • Can the space be adapted to meet the changing needs and preferences of stroke survivors? • Can the space promote practice, activity, and rest, depending on need? • Would the space allow stroke survivors to take appropriate risks based on their recovery level? And accommodate people with different abilities?	How versatile is this bedroom, on a scale from 0 to 100?	0 = a bedroom that is not at all versatile; 100 = the most versatile bedroom you can imagine.
Personal control	Think about the extent to which stroke survivors have personal control over this bedroom. • Would stroke survivors be able to make changes in the space themselves, or easily ask someone to help them do this? • Would they be able to add their own personal touches to a space? • Adjust the space to be more private or social depending on their needs and preferences? • Would a stroke survivor feel that their feelings and preferences are respected in this space? Would they feel a sense of ownership and agency?	How much personal control does this bedroom provide to stroke survivors, on a scale from 0 to 100?	0 = a bedroom that provides no personal control; 100 = a bedroom that provides the most personal control that you can imagine.
Outdoor and green spaces	Think about access to the outdoors, nature, and green spaces from this bedroom. • Is there an easily accessible door to the outside and nature? • Views of the outside and nature? • Does outdoor environment should make you feel good? • Would the outdoor area help to promote activity? • Help encourage connection to the outside community and the real world?	How good is the access to the outdoors and nature in this bedroom, on a scale from 0 to 100?	0 = a bedroom that provides no access to the outside or nature; 100 = a bedroom that provides the best access to the outside or nature that you can imagine.
Positive and stimulating environment	Think about whether this bedroom feels positive, stimulating, and fun • Would it help to promote activity and emotional well-being for stroke survivors? • Are there engaging, uplifting, attractive, and interesting destinations both indoors and outdoors to draw people out of their beds? • Is there a variety of options that cater to different interests and tastes to promote continued engagement?	How positive and stimulating is this bedroom, on a scale from 0 to 100?	0 = a bedroom that is not at all positive or simulating; 100 = the most positive and stimulating bedroom that that you can imagine.
Fundamentally important:			
Effectiveness (activity, practice, and rest), wellbeing, safety, and efficiency	Overall, the best possible bedroom for stroke rehabilitation should do the following: 1) help to optimise clinical outcomes by promoting activity (physical, cognitive, social) and rest, 2) operate efficiently, 3) maximise the emotional well-being of all users, and 4) keep all users safe.	Overall, how well do you think this bedroom achieves these things, on a scale from 0 to 100?	0 = the worst bedroom that you can imagine; 100 = the best bedroom that you can imagine.

After completing the quantitative VAS evaluation, participants’ were offered a break before viewing the next VR bedroom environment. After viewing each of the four bedrooms within the VR environment and completing the think-aloud protocol and VAS questions, participants were invited to attend a short focus group for debriefing and to allow participants to provide additional comments on the room designs, the data collection process and any other items that were not addressed during the VR sessions. The focus group questions focused on the fundamentally important objectives in stroke rehabilitation environments, namely whether participants felt that the bedrooms helped to support activity and practice, rest, safety, and efficiency (see Supplementary material). Large, printed images from each of the VR bedroom environments were available during the focus groups for the participants’ reference and to prompt discussion. The focus groups were audio-recorded.

### Data analysis

Quantitative data from the VAS responses collected within the VR user interface were automatically transferred to an online spreadsheet. First, the raw VAS scores were then analysed using descriptive statistics (median, interquartile range) and presented graphically to visually compare the extent to which each of the four bedroom designs met the predefined objectives that are important for stroke rehabilitation environment design (versatility, personal control, outdoors and greenery, positive and simulating, and overall).

Each participant’s raw VAS scores were then transformed into rankings for each of the key objectives, whereby the bedroom that received the highest score for that objective was ranked number 1 and the bedroom that received the lowest score was ranked number 4. Ties in ranking could be permitted (i.e., ranking could be tied for 2 or 3 or 4 bedrooms for one objective). If two or more of the raw VAS scores were less than +/- 5 points from each other then, these were entered as equivalent (tied) ranks. If, for example, when asked to rate the versatility of the bedrooms using the VAS, a participant gave the duet, butterfly, quartet and troika scores of 31, 91, 92, and 100 respectively, then the bedrooms would be ranked in the following order on versatility for that participant: 1 = troika, 2 = quartet, 2 = butterfly, and 3 = duet.

Participants’ individual rankings were then combined using a graph-theory-based algorithm to produce an aggregated ranking of the four bedroom provotypes for each of the key objectives [42]. This aggregation method is a robust way of combining participant’s rankings because it treats rankings as purely ordinal data and uses graph theory to extract only the ordering that the data genuinely support—i.e., allowing ties between the provotypes—rather than summing ranks or forcing a definitive ranking [42]. To check for familiarity biases, we calculated rankings for two subgroups: 1) participants who had participated in previous NOVELL events or workshops prior to completing the VR evaluation (non-novices), and 2) participants who were novices to the project.

Audio-recordings of the participants’ VR evaluation sessions (think-aloud responses and first impressions of each bedroom) and the focus groups were reviewed and all key comments were transcribed. These quantitative and qualitative data were combined to describe which values or objectives are ‘driving’ responses to each of the design options, providing actionable explanations of why some options are considered more optimal than others.

## Results

The data collected provided a broader view of what makes a ‘good stroke rehabilitation in-patient room’ with insights from both the quantitative and qualitative data.

### Speculative design outcome of provotype esquissé workshops

**Fig 4:**
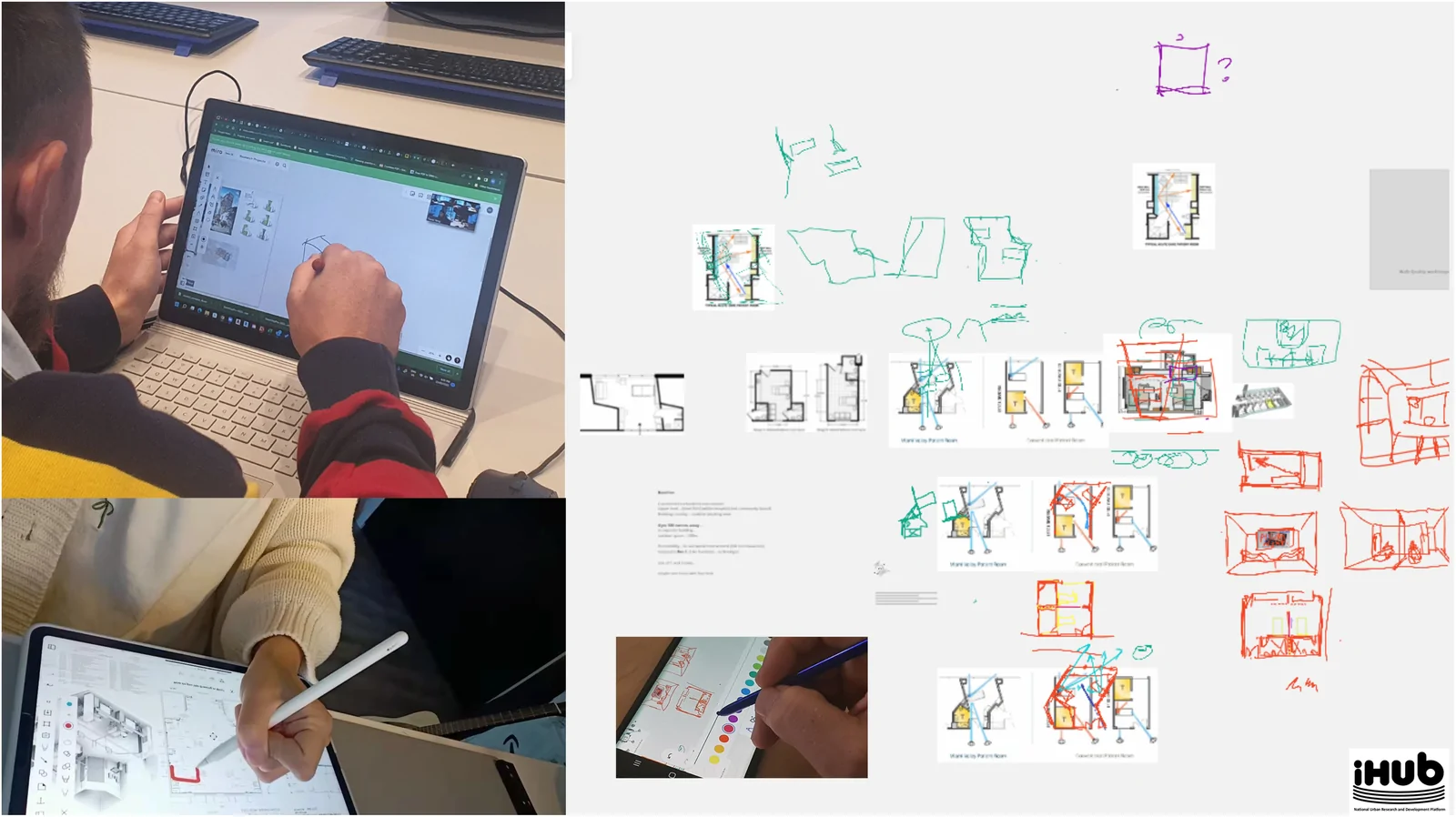
Live digital sketch design workshop. Split image showing photographs of live digital sketching of bedroom designs appearing on to Miro collaboration platform, displayed on iHUB screens during Provotype Esquissé Workshops.

**Fig 5:**
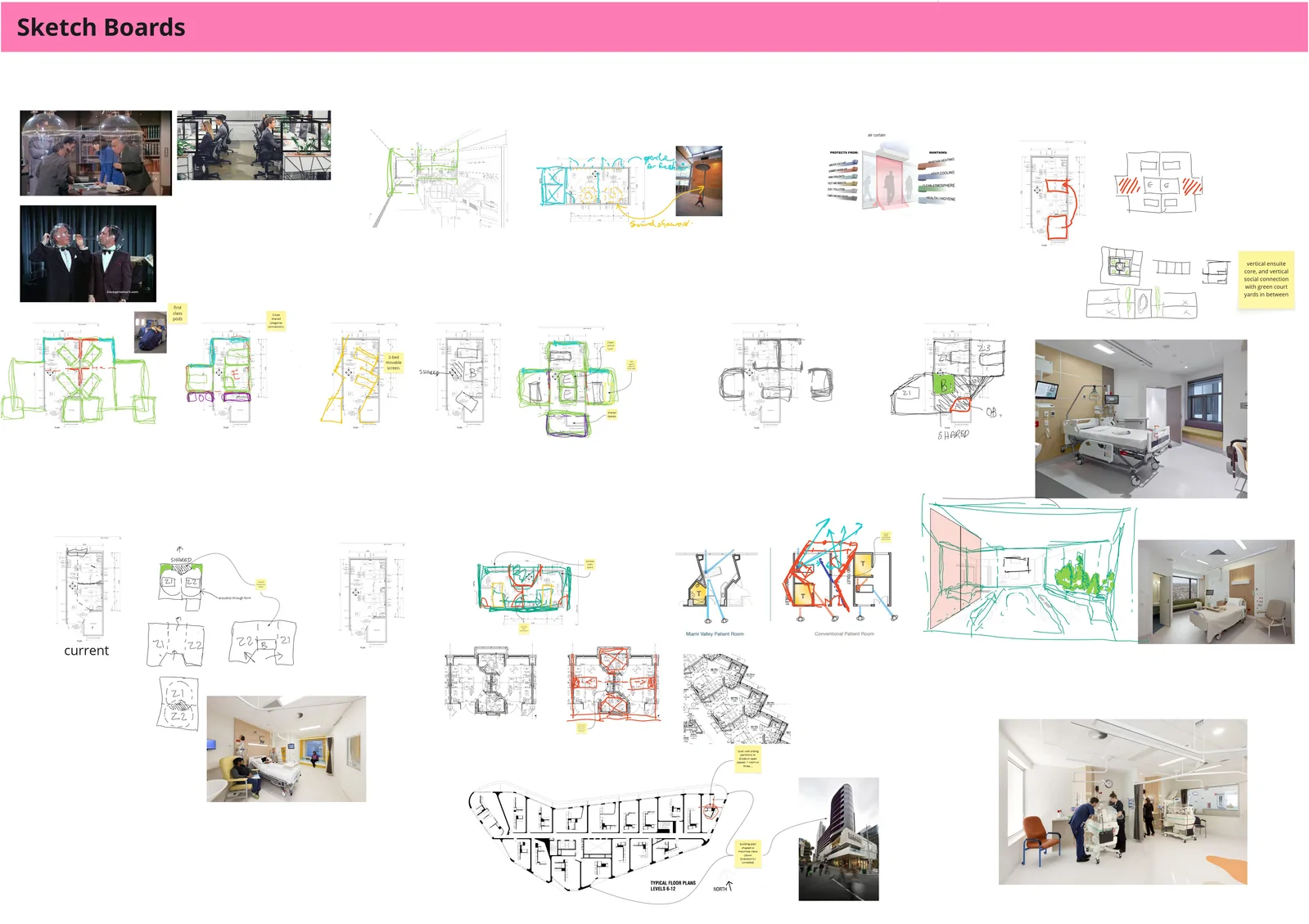
Digital sketch design. Screen grab showing digital sketching of bedroom designs including concepts of audio separation including the ‘cone of silence’ and movable office partition concepts (top left), along with room layouts that controlled levels of visual connection by angling or reconfiguring room layout shapes displayed on iHUB.

**Fig 6:**
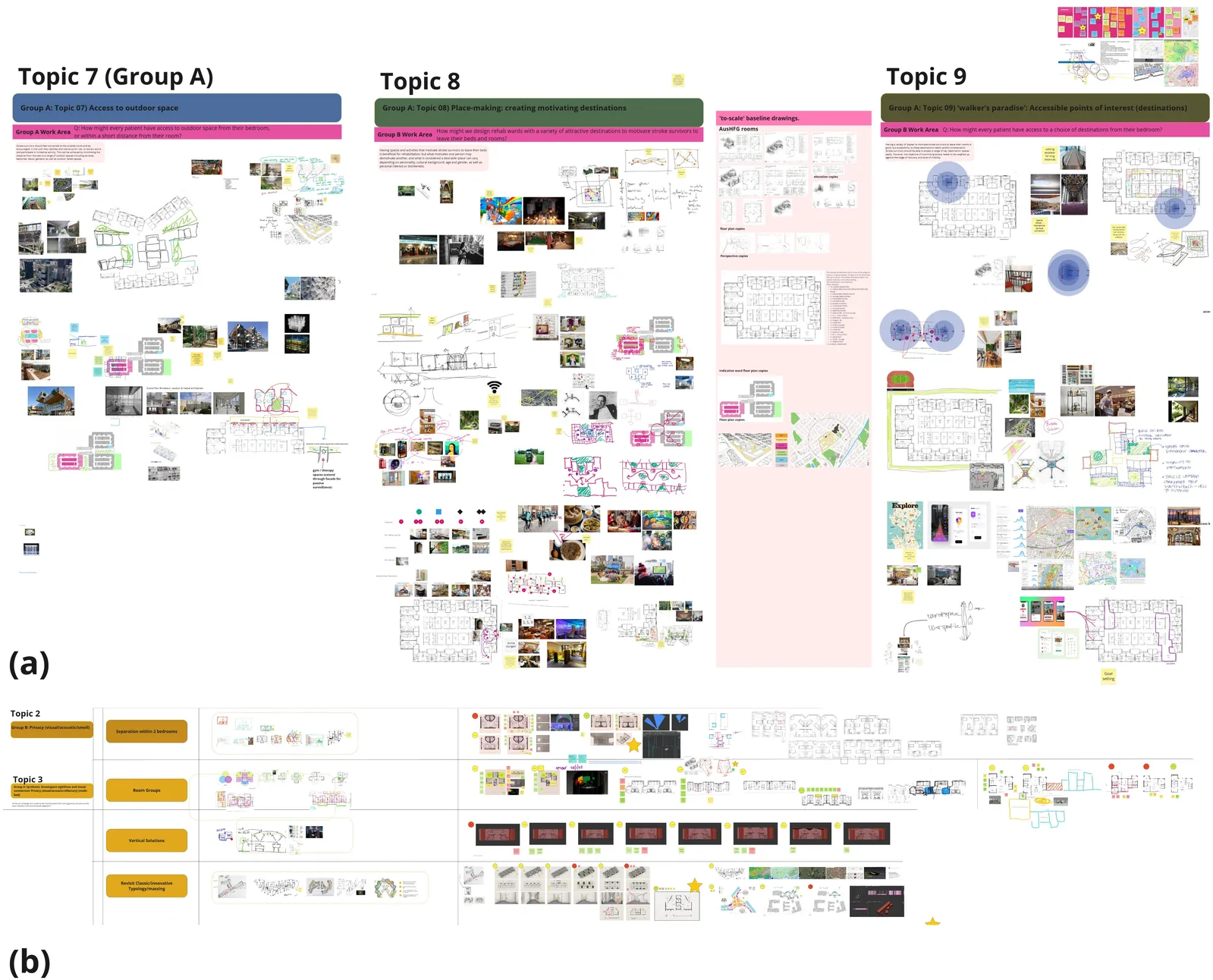
Digital whiteboard workshops. (a) Screen shot of the digital whiteboard showing the outcomes of workshops where participants were broken up into smaller groups and topics were discussed with ideas placed on to the boards in terms of notes, sketches, images and precedence. (b) The outcomes of the esquissé were then sorted based on the topic and subtopics addressed in the Esquissé Workshop (Columns 1, 2 and 3) and explored further in the Provotyping Workshops (example of these are shown in the right column).

The Esquissé workshops were divided into topics based on the Means Objectives that were determined could translate into architectural outcomes from previous workshops. These rapid design explorations took place in Swinburne University’s iHUB where Miro online collaborative whiteboard software was used as the platform to collect sketched ideas, precedent images, create mind maps, and organise information in a visual way [Fig 4, Fig 5, and Fig 6]. The groups utilised Miro to communicate, collaborate, and exchange ideas throughout the workshops as they were developed and refined. Everyone in the room and online could see the ideas and critique them when presented on the large displays in real time.

Through the rapid design process, the groups were able to input expertise from architecture, healthcare planning, health economy, way-finding, academia, technology, clinicians, and people with stroke, to generate over 500 design ideas for new stroke rehabilitation facilities. All the ideas were retained and collated and ordered on ‘Frames’ to be explored in the next stage of the design process [Fig 6].

### Design development of the ‘Provotypes’

Over a three-month period, design iterations where the various features pertaining to the ‘ideal’ rehabilitation bedroom were explored. A ‘typical’ two-bed hospital room from the findings of Yang et al.’s 2024 study on common ward and room typologies [13], along with the commonly adopted Australasian Health Facility Guidelines AusHFG guideline two bedroom unit was used as the starting point, and became the baseline comparator for the evaluations in VR.

**Fig 7:**
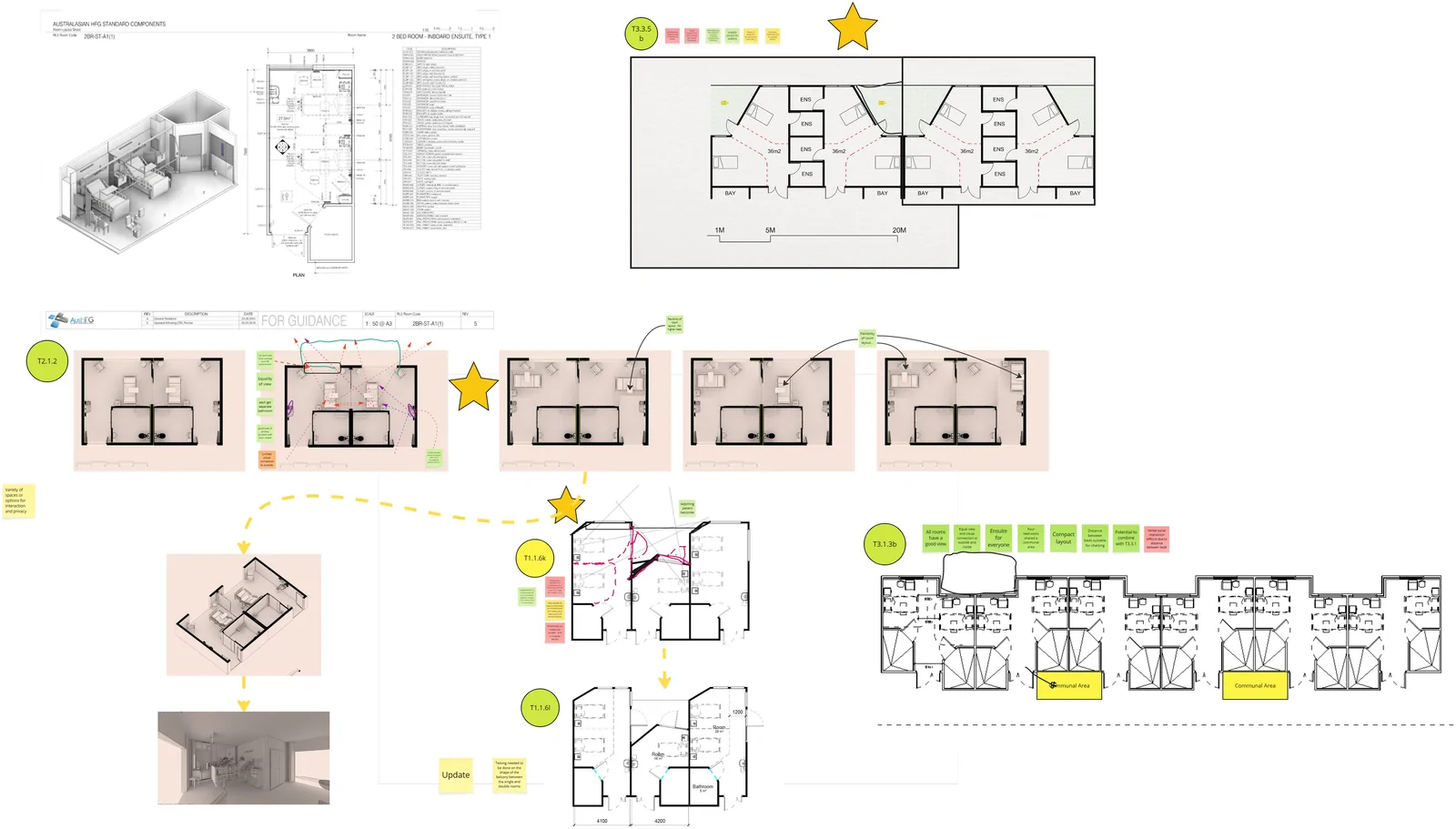
Provotyping process. Example of the provotyping process to generate options for the bedrooms.

The design team drew up various provocative prototype (provotype) hybrid-two bed room configurations and placed them on a Miro board. These designs were developed directly focusing on the key means objectives mentioned above [22,23], and were allowed to push designs beyond what was ‘normal’ in term of size, shape and configuration, as well as established design principles incorporated into the AusHFG guidelines two-bed room layout. The designs were critically reviewed by the design team, and stakeholders of the project, with comments provided. Shortlisted options were progressed to the next step which was 3D modelling and white-card rendering (rendering without colour, materials and textures) [Fig 7].

A color-coded system of sticky notes and dots to identify and track the evolution of the preferred options was employed. Green sticky notes highlighted positive feedback for ideas to develop further, while red notes pointed out features that were deemed negative or should be eliminated. Three coloured dots were used to assess design options: red indicated rejection, yellow suggested potential with some adoptable ideas, and green signified strong options that should be further developed. A gold star marked design ideas that were selected for inclusion in a provotype room [Fig 7].

Designs were generated based on the preferred spatial features highlighted by participants from the esquissé workshops. These features were emphasised in varying degrees in each of the provotypes and were proposed to respond to one or more of the objectives.

Table 1 below breaks down the degree of emphasis on each means objective feature that occurred at each bed within the two-bed bedroom provotype. Four final designs were selected and further developed: The Duet (baseline), The Butterfly, The Quartet, and The Troika. For example, access to outdoor spaces and greenery (predefined means objective) was prioritised for both beds in the Butterfly, Quartet, and Troika rooms. This included physical access to the outdoors, but also visual connection from within the room and from bed (i.e., window views to outside). In the Troika room, the window view for Bed 2 was obscured if the patient decided to close their own privacy curtain, hence the reduced emphasis on ‘access to green space’ for this bed.

The amount of emphasis for each actionable solution, or design feature, for each of the beds within the bedroom were rated at a scale of 0–100 by the design consultants and lived experience group in review workshops and is expressed in a heat map scale. Bed 1 is located nearer to the entry door whilst bed 2 is located nearer to the façade of the building (and window). The selected provotypes were all given names that were considered pleasant, but non-descriptive so that participants did not have any preconceptions of the design.

The provotypes were further refined with fit out, furnishing, and technology items highlighted from the esquissé workshops, first in plan drawings then in 3D BIM modelling and rendering. The provotype room models were transferred into a game engine where a further refinement of the equipment and material renders was done, as well as animation of the key design features. The audio description of the room and the custom VASs were also added.

### Duet (baseline comparator)

**Fig 8:**
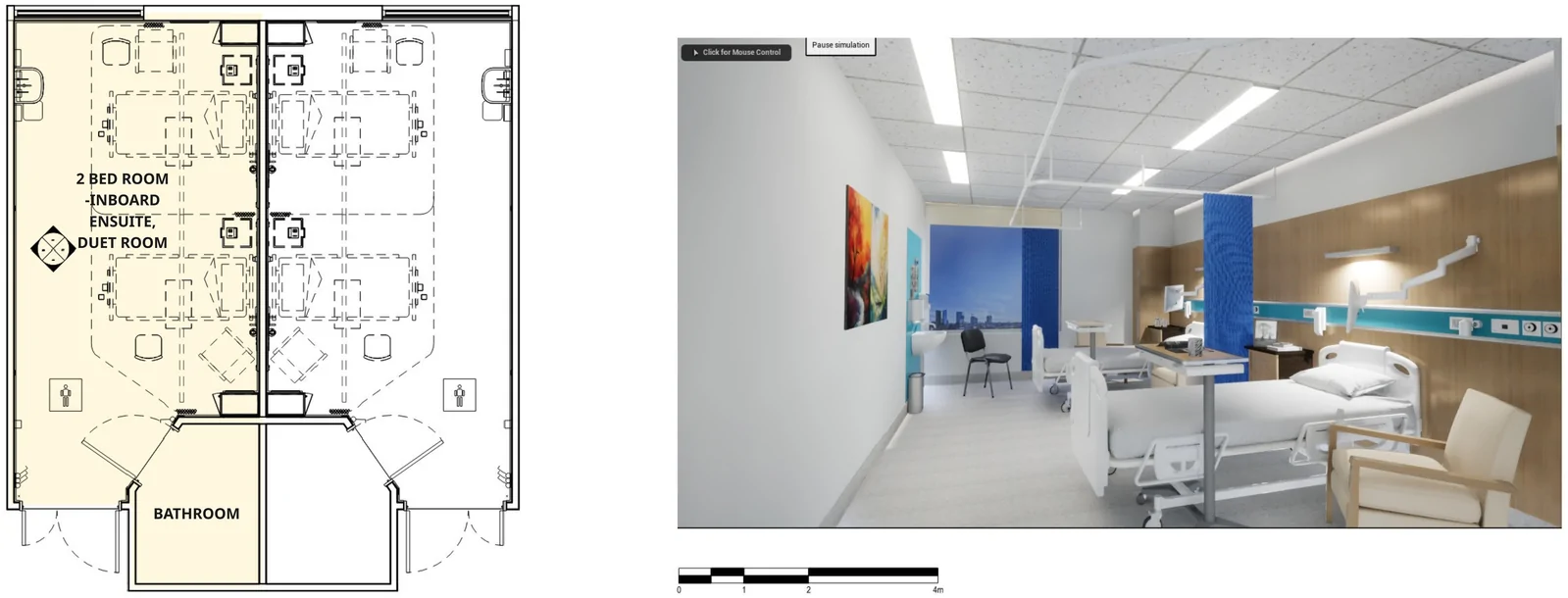
The duet room (Baseline). Colour codes indicate: Extent of Bedroom (orange) and Adjacent Room (white).

The design for the Duet was based on the AusHFG standard component for a typical two bed room with a shared ensuite [Fig 8] [43]. We chose this as the baseline type for comparison with the other three designs.

The finishes to this room were designed to provide a pleasant and cheerful aesthetic to make residents as comfortable as possible in the environment. The fit out to the room was standard hard wearing, easy to clean materials that were typical to acute hospital rooms. Items that deviated from the basic room were a timber veneer feature wall with highlight strip lighting, feature colours to the handwash area and the provision of hung artwork to the walls. To provide a ‘clean’ contemporary aesthetic, the background colour was rendered as white painted plasterboard for both walls and ceilings.

### Butterfly

**Fig 9:**
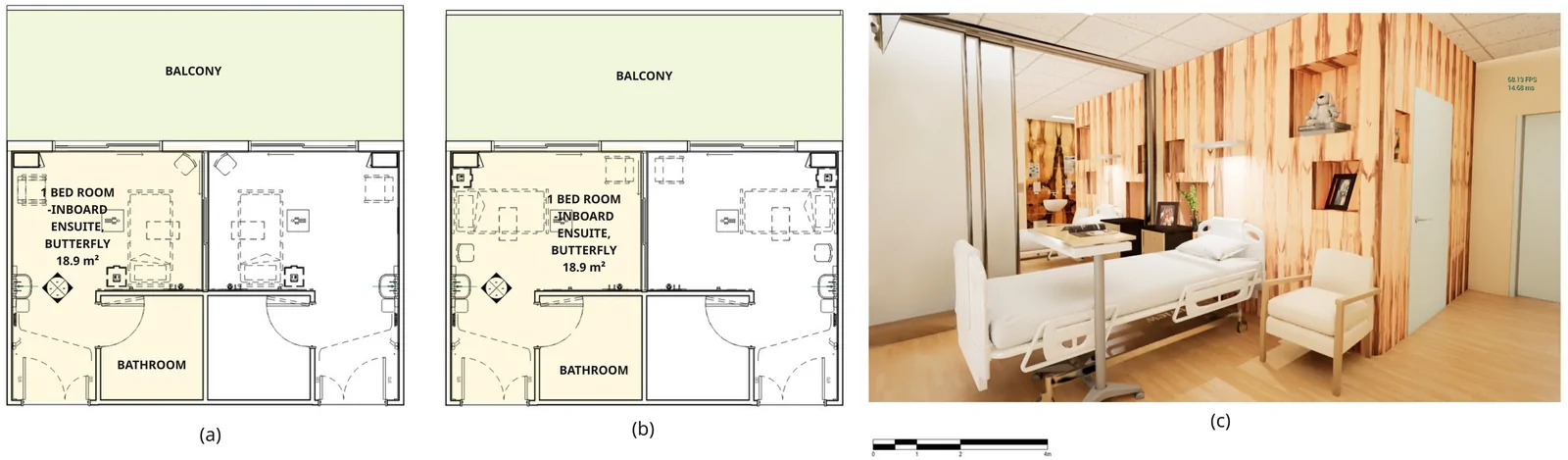
The Butterfly room provotype. Left: Plan of the Butterfly Room Provotype in two configurations: (a) bed positioned against the ensuite wall to facilitate patient-patient interaction; (b) beds positioned against the fixed dividing wall to enhance visual access to the winter garden. Colour codes indicate: Extent of Bedroom (orange), Adjacent Rooms (white), Courtyards and Outdoor Spaces (green). (c) Screenshot of the VR scenario showing the Butterfly Room with the frosted glazed, soundproof sliding partition wall in the open position.

The Butterfly rooms are double rooms with an ensuite for each occupant [Fig 9]. These rooms can be converted into single rooms (with two ensuites) by closing a frosted glazed soundproof sliding partition wall. The configuration of the room can also be adjusted by placing the bed against the ensuite wall or the fixed dividing wall. The Butterfly room could feasibly be achieved through retrofit or renovation of many existing hospitals with a standard repetition of adjoining single-bed rooms. The former configuration allows for greater privacy from the corridor whilst the latter allows for better supervision by staff from outside of the room.

The Butterfly room design incorporated almost all of the desired features Table 2. The sliding frosted partition wall allows the person with stroke to choose to interact with another patient or not. Operation of the wall is by voice-operated remote control, or a large button located within reach of the bed, allowing for accessible operation, and requires the consent from both patients as to which arrangement they prefer.

**Table 2:** Room provotypes and their design emphasis.

Feature	Duet		Butterfly		Quartet		Troika		
Bed 1 & Bed 2	Bed 1	Bed 2	Bed 1	Bed 2	Bed 1	Bed 2	Bed 1	Bed 2	
Visual Connection / Isolation	*100*	*70*	*100*	*100*	*100*	*80*	*100*	*70*	
Acoustic Connection/ Isolation	*0*	*0*	*100*	*100*	*0*	*0*	*60*	*60*	
	*0*	*0*	*0*	*0*	*0*	*0*	*60*	*60*	
Access to green space	*0*	*0*	*100*	*100*	*100*	*100*	*100*	*90*	Scale
View to the external from all beds	*70*	*20*	*100*	*100*	*100*	*100*	*100*	*90*	*100*
Supervision from staff	*70*	*70*	*100*	*40*	*70*	*90*	*90*	*70*	*90*
Secure Personal Storage	*25*	*25*	*80*	*80*	*100*	*100*	*25*	*25*	*80*
Display space for personal items	*10*	*10*	*100*	*100*	*100*	*100*	*10*	*10*	*70*
Electronic aids, remote control of immediate surroundings	*25*	*25*	*100*	*100*	*25*	*25*	*100*	*100*	*60*
Interchangeable bed arrangement	*0*	*0*	*100*	*100*	*0*	*0*	*0*	*0*	*50*
Space for Visitors / Overnight Stay Bed	*0*	*0*	*80*	*80*	*100*	*100*	*0*	*0*	*40*
Space for Therapy / Exercise	*0*	*0*	*90*	*90*	*100*	*100*	*25*	*25*	*30*
Space for storing wheelchair/mobility aid	*10*	*10*	*60*	*60*	*100*	*100*	*10*	*10*	*20*
A/V Connection to outside world	*100*	*100*	*100*	*100*	*100*	*100*	*100*	*100*	*10*
Electronic Rehabilitation Progress Charts	*70*	*70*	*100*	*100*	*100*	*100*	*70*	*70*	*0*

Other features include timber veneer to the external of the ensuite walls, with nooks and shelves in the wall for placing personal items and memorabilia, different colour schemes to the walls, additional space for a desk, in-room therapy or a daybed for visitors. An outdoor winter garden can be accessed from the sliding doors at the façade side of the room.

### Quartet

**Fig 10:**
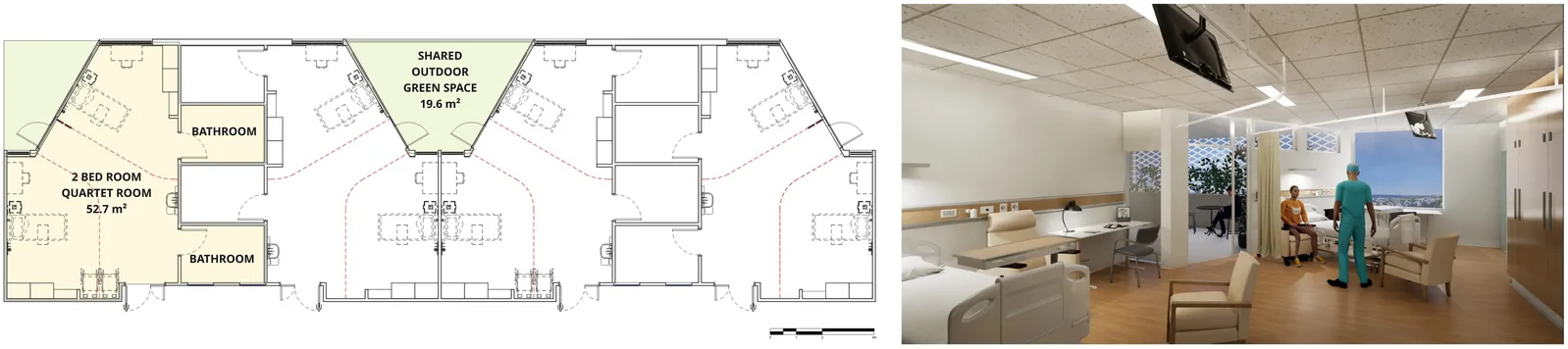
The quartet room provotype. Left: Plan of the Quartet Room Provotype. Colour codes indicate: Extent of Bedroom (orange), Adjacent Rooms (white), Courtyards and Outdoor Spaces (green). Right: Screenshot of the Quartet Room VR scenario.

The Quartet rooms are pairs of two-bed rooms that affords an ensuite to each occupant [Fig 10]. The rooms are laid out in pairs with the ensuites forming the division between the rooms. Each room has a shared outdoor space (on the non-ensuite side) with the other neighbouring room. For the quartet arrangement to work there needs to be at least two rooms side by side.

Like the Butterfly room, the Quartet includes many of the desired design features (Table 1), but the Quartet has more space for storage and circulation, and does not have the level of acoustic separation, bed position flexibility, or remote control of features that are present in the Butterfly room. Each bed has its own private secure storage cupboard and a desk. There is a storage nook for mobility aids next to the entry door. There is ample space between the beds for in-room exercise therapy, visitors, an extra day bed for family to stay. Visual privacy between beds was afforded by curtains that enclose each individual bed area. There is easy access from either bed to the outdoor balcony, and view to the outdoors from both beds. The room background colour is white for both walls and ceilings, with a warmer toned floor and timber veneer joinery.

### Troika

**Fig 11:**
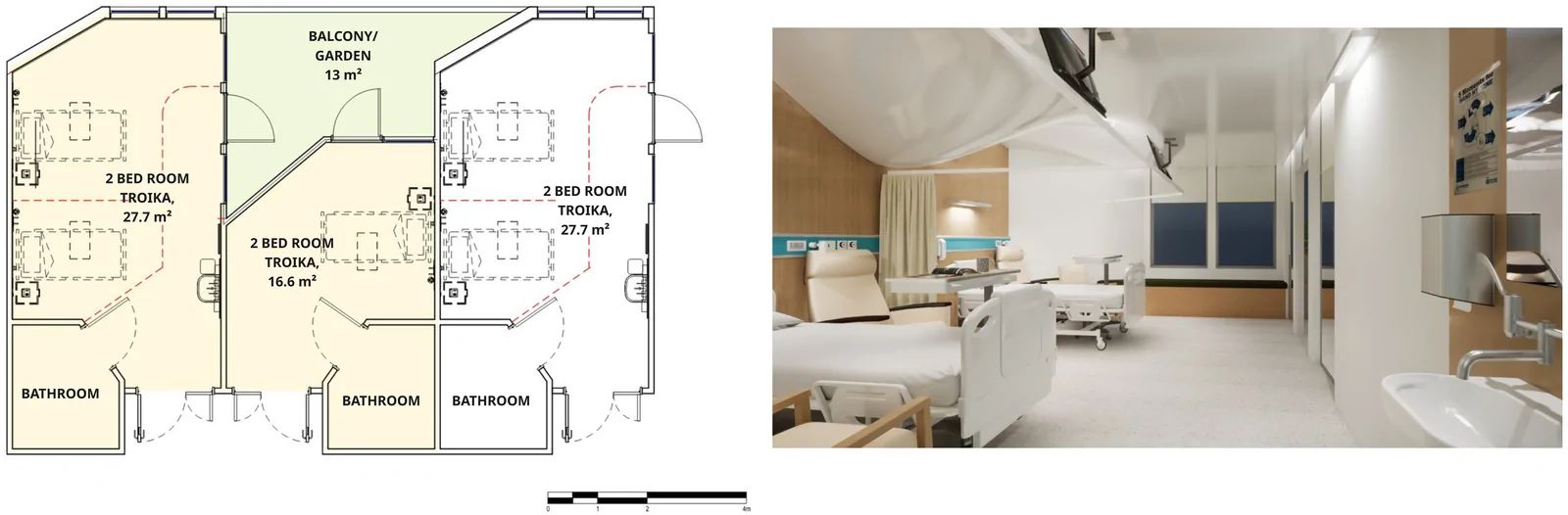
The trioka room provotype. Left: Plan of the Trioka Room Provotype. Colour codes indicate: Extent of Bedroom (orange), Adjacent Rooms (white), Courtyards and Outdoor Spaces (green). Right: Screenshot of the Troika Room VR scenario. The adjustable acoustic ceiling is shown in the ‘acoustic separation’ arrangement.

The Troika is based on the arrangement of one two-bed room paired with a one-bed room, with both rooms having equal access to a shared private balcony [Fig 11].

The Troika includes fewer of the desired design features than the Quartet or the Butterfly, but has greater emphasis on patient control over acoustic connection and separation (Table 1). All beds in the troika have views to the outside. The occupant of the inner bed in the two-bed section of the Troika is able to look outwards as there is extra window space afforded by the adjoining room, which is a smaller single bedroom that is set back further from the façade. The troika also had a bay window at the façade, providing seating or a bed for family. The Troika rooms have an adjustable acoustic ceiling arrangement that allows patients to regulate their acoustic privacy needs; the shape of the ceiling between the two beds can be adjusted via button-press or voice activation to be either convex (providing acoustic separation between the beds) or concave (providing acoustic separation between the beds). A desktop analysis on the effectiveness of the acoustic ceiling system was done to determine which configuration worked best prior to integrating it into the Troika design.

### Room sizes

The sizes of the rooms ranged from 26m2 to 52.7m2, with varying ensuite and outdoor spaces [Table 3]. The size of the rooms was generally consistent with AusHFG guidelines with Duet room matching double bed room exactly, while others closely matching either single or double sizes, other than the Quartet, which was larger than the standard rooms. Size was noted by most of the participants in the evaluation sessions, indicating the effectiveness of size/spatial perception provided in VR.

**Table 3:** Floor area comparison between the four provotype rooms.

Provotype Room Name	Interior	Ensuite	Exterior (Balcony)
**Duet**	26.0 m^2^	4.0 m^2^	0 m^2^
**Butterfly**	18.9 m^2^ x 2 *	5.6 m^2^ x 2	28 m^2^
**Quartet**	52.7 m^2^	7.0 m^2^ x 2**	9.8 m^2^
**Troika**	27.7 m^2^ + 16.6 m^2^ ***	4.9 m^2^ x 2	13 m^2^

*The butterfly room comprises two single bedrooms with a soundproof partition wall that can be opened to create a single two-bed room. It affords one ensuite per bed.

**The outdoor space in the Quartet is shared between two rooms, the area associated with each room is half of shared outdoor space available for the patient.

***The Troika room arrangement consist of one two bed room and one single bed room with a shared outdoor space. The two bed room has a shared ensuite.

### Outcome of the VR Data Collection Workshops

Data were collected during workshops conducted at three different locations in central Melbourne, Australia, over the course of four days. Each participant typically spent about 30 minutes in total to view and evaluate the four bedroom options. Of the 21 participants recruited, one did not participate due to internet outage, leaving a final sample size of 20 participants, with representation from each of the target expert stakeholder disciplines (Table 4).

**Table 4:** Table describing participant demographics.

Variable		Participants, *n* = 20
Expert stakeholder discipline^a^, *n* (%)		
	Person with stroke	5 (25)
	Clinician	7 (35)
	Architect or designer	8 (40)
	Researcher	3 (15)
	Policy or health services	4 (20)
Number of years post-stroke^b^, mean (range)		14.8 (8-30)
Gender, *n* (%)		
	Female	13 (65)
	Male	6 (30)
	Non-binary/other	1 (5)
Age, *n* (%)		
	18-25 years	1 (5)
	26-35 years	2 (10)
	36-45 years	6 (30)
	46-55 years	5 (25)
	56-65 years	5 (25)
	66 + years	1 (5)
Previously participated in NOVELL events^c^, *n* (%)		
	Yes	13 (65)
	No	7 (35)
Used VR in the past, *n* (%)		
	Never	8 (40)
	Once or twice	11 (55)
	Many times	1 (5)

a Some participants belonged to more than one discipline (e.g., person with stroke who is also a clinician).

b Only applicable to the participants who had had a stroke.

c Participants who had not previously participated in events or workshops with the NOVELL project were considered novice to the project.

### VAS responses and aggregated rankings

**Fig 12:**
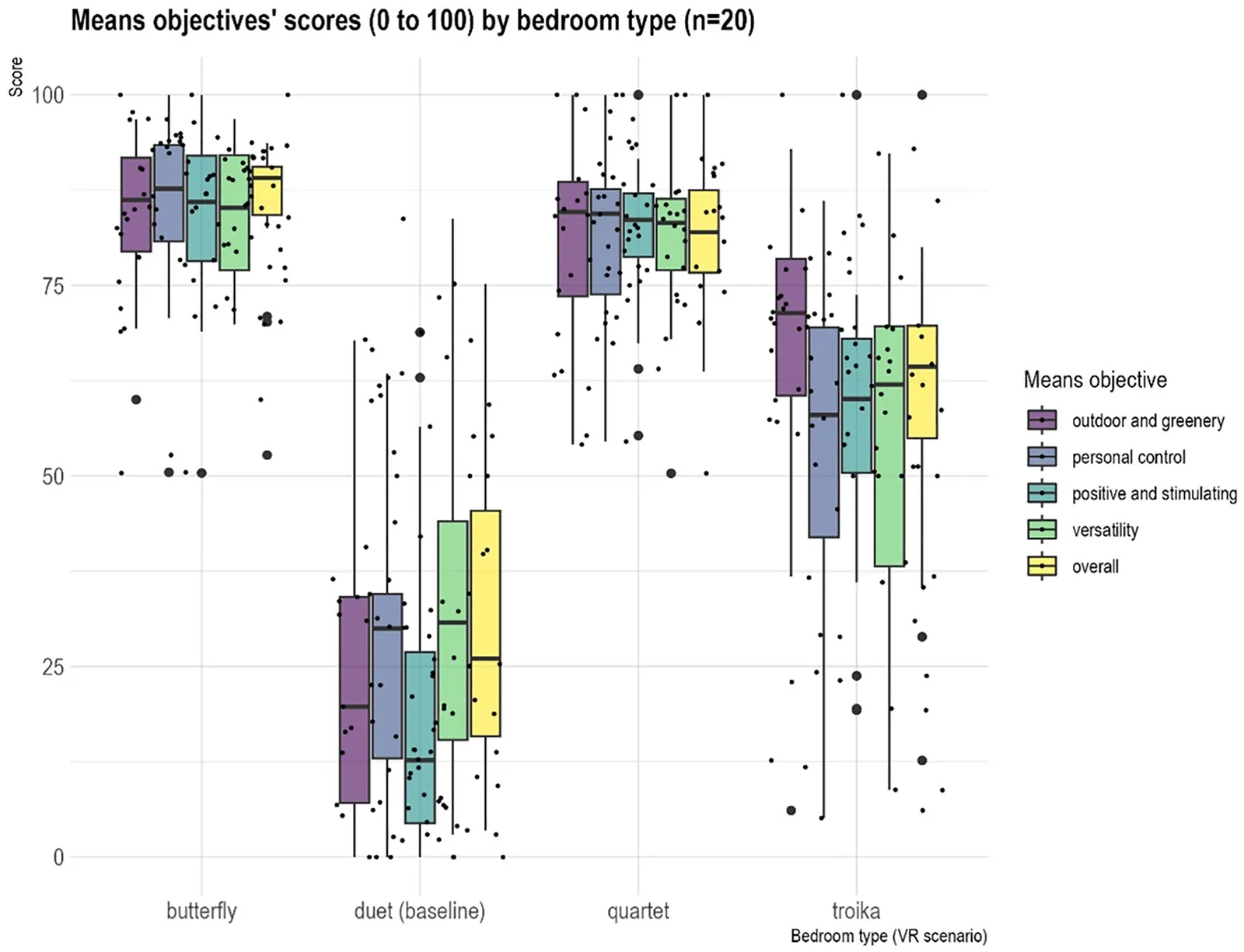
VAS Raw Scores for each of the key objectives for each of the four provotype bedrooms, collected within the VR room experiences.

The butterfly room achieved the highest median VAS scores compared to the other bedroom design options for all of the key objectives, followed by the quartet and the troika, with much lower scores given to the baseline duet design (Fig 12). There was, however, a big range of scores given to the duet and the troika (i.e., large interquartile range) for all of the key objectives, whereas participants appeared to be more in agreeance about the butterfly and quartet (smaller interquartile range) (Fig 12).

According to the aggregated rankings, the butterfly and quartet rooms were tied at top ranking for all of the key objectives listed in Table 1 (versatility, personal control, access to outdoors and greenery, positive and stimulating environment, and overall achievement of encouraging activity, rest, wellbeing, safety, and efficiency), while the duet had the lowest ranking for all the objectives. The butterfly and quartet provotypes remained in the top two rankings, and the duet in the lowest ranking, for all objectives for both the novice and non-novice participant subgroups. Details of the aggregated rankings are included in supplementary material.

### Participant’s verbal feedback and focus group responses

The comments recorded during the evaluation workshops and focus group sessions offered additional insights into both the design of the rooms and the overall design process. Some of these comments are provided in Table 5. Comments from the participants reinforce the quantitative data and the means objectives. Preferences for the designs were based on the participants’ background and what they perceive as important. It was noticeable that the Duet, which was based on a baseline two bed room received minimal positive comments. The participants’ backgrounds had some bearing on the types of comments received. Most of the participants were able to identify that the Duet was based on a standard two bed room, and did not provide any significant comments on how it addressed the means objectives.

**Table 5:** Participant comments.

Objective	The quartet	Troika	Butterfly	Duet
**Personal control**	“there’s a significant enough distance between the beds that you could still have privacy if you wanted to say something personal” Stroke survivor “Having the hand basin and bathroom near the door means that nurses could wash their hands in the middle of the night without disturbing you, or cleaner could come in and clean the bathroom” Stroke survivor		“It gave you the option of sharing the view with your neighbour, or closing it off” Stroke survivor “I love the privacy, but also the option of opening up to the person next to you. You don’t know who might be next to you – you might become best friends!” Stroke survivor	
**Versatility**		“It’s incrementally changed from the typical two-bedders. I find it very clever… [it could] definitely work in a refurb” Health management	“you could have the view, have the bathroom, have the circulation, have the space, have the desk… you could get fresh air…” Stroke survivor	
**Outdoors and green**			* “that long [balcony corridor] would be a fabulous place to walk up and down because it’s measurable… you can measure your progress… I can really see it promoting people walking, and having something to look at while you’re walking, but still being inside if it was raining” stroke survivor	
**Positive and stimulating**			“I really love the wood panelling, the nooks, it does look like a little hotel room. And the little balcony, being able to go out there. It’s just nice and bright.” Stroke survivor	
**Overall**	“From an architectural point of view, the quartet, I like it the most, really spacious” architect “it gives privacy – with more space between beds, I like that – and there’s also more space to get out and move around” clinician “in my ideal world I would choose the quartet. More spacious, more natural light.” clinician		“If I’m ever in hospital again after a stroke, put me in the butterfly room” lived experience “lots of privacy if you want it. You can close it off or open it up, and still get good access to balconies” stroke survivor. “The butterfly looks like a little hotel. I would go out on that balcony and do some standing exercises there.” Stroke survivor “I’d go to the butterfly and I’d stay there for a month before I’m ready to go home, thank you! [laughs]” Stroke survivor “This would find me the most optimal chances of getting better” Stroke survivor	
**Activity**	“It’s more open. You can move around and get out between the beds.” Stroke survivor		“that long corridor [outdoor] would be a fabulous place to walk up and down because it’s measurable… you can measure your progress… I can really see it promoting people walking, and having something to look at while you’re walking, but still being inside if it was raining” stroke survivor “Waking up in that room I’d think ‘right, I’m up, let’s get going! I’m ready to start the day!” Stroke survivor	

Analysis of comments and the Means Objective used to generate the room types that the participants responded to. Some of the comments relate to several means objectives, these are highlighted with an asterisk*.

## Discussion and conclusion

### ‘Single’ versus ‘multi’ versus ‘hybrids’

The methodology developed for this study was anchored in a values-based, collaborative approach, stakeholders developed the objectives to inform design, contributed to design, and evaluated the designs in virtual reality according to how well the designs met the predefined objectives. The methodology proved an effective means of stakeholder evaluation of design, with two of the designs clearly ranking higher on all predefined objectives. The preliminary findings from this study challenge the long-standing binary debate between single and multi-bed rooms in stroke rehabilitation by demonstrating the viability of hybrid solutions. This finding warrants clinical and post-occupancy evaluations to establish therapeutic impacts of hybrid bedroom designs. Previous research indicates that traditional single and multi-bed configurations each have inherent limitations [10,16,17,36]. Single rooms excel in privacy and environmental control but may isolate patients, while multi-bed rooms foster social interaction but often compromise rest and personal space. The hybrid provotypes developed in this study, particularly the *Butterfly* and *Quartet* designs, were perceived by respondents to have successfully integrated the benefits of both room types while mitigating their drawbacks.

Based on the initial findings, the *Butterfly* room presented as the most preferred option, largely due to its adaptable sliding partition, which allowed the person with stroke to modulate privacy and social interaction dynamically. Stroke survivors particularly valued the ability to control their environment, with one participant remarking, ‘If I’m ever in hospital again after a stroke, put me in the Butterfly room’. This design also addressed concerns around noise and light disturbances, which were frequently cited as major drawbacks of conventional multi-bed rooms. The inclusion of private ensuites and access to outdoor spaces further enhanced its appeal, reinforcing the importance of restorative environments in rehabilitation. The *Troika’s* acoustic ceiling treatment appeared to have a positive impact, though not to the extent of the *Butterfly*’s sliding partition. This may be due to the familiarity of a sliding door as compared with a mechanically deforming ceiling. Positive responses to both highlight the importance of integrating smart materials and adaptive technologies into rehabilitation environments.

The *Quartet* design, while more spatially demanding, received positive responses for its balance of shared and private zones, offering people with stroke both social engagement and personal retreat spaces. Clinicians noted its potential to facilitate in-room therapy and family visits, while architects highlighted its efficient use of space despite its larger footprint. However, cost implications must be considered, as the *Quartet*’s expanded floor area may pose feasibility challenges in resource-constrained healthcare settings. It is important to consider both up-front capital costs as well as longer term cost benefits of more effective rehabilitation. This aspect of short and long-term costs is studied in detail elsewhere by Kerr et al., [12], with conclusions suggesting that higher capital cost for better rehabilitation designs can be considerably more cost effective in the longer term.

In contrast, the baseline *Duet* (a standard two-bed room) performed poorly across multiple criteria, underscoring the inadequacies of current normative designs. Participants criticised its lack of privacy, noise issues, and inflexibility, reinforcing existing literature on the shortcomings of conventional multi-bed configurations in long-term rehabilitation.

The findings of this study underscore the importance of patient room designs that integrate a balance of personal privacy, comfort, perceived and visual connection to the natural environment (including access to daylight and green spaces), physical therapy, safety, and opportunities for social interaction with fellow patients or visitors. Key factors identified were the occupant’s ability to exercise personal control over their environment, and the degree of social engagement.

Our results support the need for multi-bed rooms that provide a mix of personal privacy and amenity, the feeling connectedness to the outdoors (access to daylight and green spaces), safety and the opportunity to interact socially with a fellow patient or with visitors. The ability for the occupant to personally control their connection to their environment and amount of interaction with others was also important. The room layouts that exhibit all of these factors were the ones that the participants felt met the objectives that had been predefined as important in stroke rehabilitation environments to maximise clinic outcomes, wellbeing, safety, and efficiency. These findings tie together the observation of others [19,20,44–46], where the need to address environmental and social factors in stroke recovery bedrooms must be met to ensure better recovery outcomes [9].

Evaluation of designs in Virtual Reality marks a significant methodological evolution in Evidence-Based Design (EBD). Traditionally, EBD in healthcare relies on retrospective data, analysing existing facilities to determine what has historically worked. While this study reinforces established EBD principles, such as the restorative power of nature and the necessity of acoustic control [20,36], it expands the framework by using Virtual Reality (VR) as a tool for ‘prospective evidence generation’. By immersing stroke survivors, clinicians, and designers in ‘provotypes’ before they are built, we move beyond merely applying existing evidence to actively generating fresh, quantifiable data based on the lived experience of future users. This approach bridges the gap between theoretical design and clinical efficacy; it allows for the empirical measurement of ‘environmental affordances’, such as the specific psychological impact of a sliding partition versus a standard curtain, through real-time stakeholder feedback. Consequently, these findings do not just advocate for a new room layout, they provide a validated model for how hybrid environments can be engineered to meet the complex, often conflicting, neuro-rehabilitative needs of privacy and social stimulation simultaneously. Clinical and longitudinal evaluation is needed to determine whether these layouts meet neuro‑rehabilitative needs in practice.

### Key methodological insights

Our method proved instrumental in bridging the gap between stakeholder input and architectural realisation, demonstrating how a cooperative, multidisciplinary approach can translate lived experiences, operational insights, and functional requirements into tangible design solutions. By systematically engaging stroke survivors, clinicians, architects, and policymakers at every stage, from initial ideation to virtual testing, our method ensured that the resulting provotypes were not only innovative but also rooted in real-world rehabilitation needs. The success of this approach highlights the value of co-design in healthcare architecture, particularly for complex environments like stroke rehabilitation facilities, where spatial design is particularly relevant to patient behaviour and wellbeing.

A key strength of this study was its integration of diverse methodologies, including values-based briefs, rapid prototyping, virtual reality (VR) simulations, and Multi-Attribute Evaluation with multiple stakeholders. The ‘Provotype Esquisse Workshops’ were particularly effective in fostering creative, boundary-pushing ideas while maintaining a focus on practical problem solving and usability. These workshops encouraged speculative thinking, allowing stakeholders to explore radical solutions, such as adjustable partitions and hybrid room configurations, that might otherwise have been dismissed in conventional design processes. The iterative nature of these sessions, combined with real-time digital collaboration tools and enabled rapid refinement of concepts into viable provotypes.

The use of Building Information Modelling (BIM), VR, and game engine technologies provided further confirmation of the effectiveness using a robust framework for evaluating design performance before physical implementation expanding on previous work [47–50]. Immersive VR testing allowed stakeholders to experience and critique virtual room provotypes in real-time, offering both quantitative metrics (VAS ratings) and qualitative feedback ‘think aloud’ commentary, [39,50]. This approach not only validated design decisions but also revealed subtle user preferences. These included the importance of acoustic control and visual access to outdoor spaces, an issue that might have been overlooked in traditional purely visual design reviews.

Critically, this study demonstrated that stroke survivors and clinicians can effectively engage with VR environments to assess rehabilitation spaces, opening new possibilities for cost-effective, user-informed design iterations. By identifying optimal design features early in the process, healthcare providers can avoid costly post-construction modifications and create environments that actively support recovery.

### Limitations and suggested further research

While this study demonstrates the potential of hybrid room designs in stroke rehabilitation, several limitations must be acknowledged. The integration of diverse design concepts into functional provotypes requires specialised expertise in architecture, virtual reality (VR) modelling, game engine technology, and specialised data analysis, which may limit widespread adoption without adequate training and resources. Additionally, some proposed solutions, such as adjustable partitions and expanded spatial layouts, may involve higher initial capital costs. A comprehensive economic analysis is needed to evaluate not only capital expenditure but also long-term benefits, including operational efficiencies, staff retention, and improved patient outcomes such as reduced long-term disability and increased employability. As mentioned above, while preliminary studies in this area do suggest potential overall cost benefits of these designs outweigh initial capital outlay as described in the 2025 study by Kerr et al. [12], ideally, built versions of these designs should be studied with post occupancy evaluation, and longitudinal studies on patient outcomes to provide stronger evidence of any benefits.

This study focused primarily on new construction, but there is potential to further study and apply the methodology to retrofitting existing buildings. The butterfly room, appeared particularly promising in this respect as its dimensions and layout fit within common construction grid and existing side-by-side single hospital room layouts.

The study’s findings may also be influenced by contextual factors such as geographical location, cultural preferences, and healthcare system constraints. This work was conducted in Australia, referring to Australasian design guides, and building constraint conditions. Expanding this research to include international settings could provide valuable insights into how these designs perform across different healthcare environments. In addition, the relatively small participant sample could be expanded to a larger cohort to increase representativeness and validity.

While we included a mix of participants familiar with the project and those who were unfamiliar with the project (novices), it should be noted that participant recruitment relied on existing collaborative networks. None of the participants who were familiar with the NOVELL project (non-novices) had seen the final provotypes prior to evaluating them in VR, but some were involved in the initial speculative design esquisses. The network-based recruitment and involvement in earlier stages of the project introduces potential bias, including research-team proximity and familiarity bias which may skew results in favour of designs or concepts that participants were already familiar with. Participants did not, however, favour the duet design, despite this design arguably being the most familiar as it drew heavily from currently Australian guidelines and norms. Reassuringly, we did not observe any notable differences in responses between participants who were familiar with the project (non-novices) versus those who were unfamiliar (novices); aggregated rankings of the bedroom provotypes for both subgroups placed the butterfly and quartet provotypes in the top two. In future expansion of this research, however, wider reaching recruitment methods should be included to ensure a wide range of opinions, including people who are not familiar with the priorities and objectives that were defined in the NOVELL project.

Although VR proved effective in evaluating design preferences, it has inherent limitations in fully replicating real-world sensory and spatial experiences, as noted by Yang et al. [50]. To address this, the highest-scoring provotypes, particularly the *Butterfly* and *Quartet* designs, could be developed into physical mock-ups for further testing. This would allow for more nuanced feedback on usability, acoustics, and material performance.

The success of the methods used in the NOVELL project in this context highlights its potential applicability to other healthcare design challenges where person-centred care and multidisciplinary collaboration are critical, such as dementia care and paediatric rehabilitation.

Finally, the data generated in this study, particularly regarding spatial flexibility, privacy, and therapeutic adjacencies, can serve as a foundation for evidence-based design guidelines. These guidelines should inform the development of future neurorehabilitation facilities, whether in new constructions or refurbishments, ensuring that architectural solutions align with the evolving needs of stroke survivors and healthcare providers. By addressing these limitations and pursuing further research, the field can move closer to optimising rehabilitation environments for wellbeing and recovery.

## Conclusion

Our findings indicate the potential for using hybrid rooms to achieve the best of both Single and Multi-Bedroom types in a hospital setting. To address the Single vs. Multi-Bedroom debate in stroke rehabilitation, we co-designed and evaluated hypothetical hybrid room designs aimed at meeting objectives identified by stakeholders. We integrated input from multiple stakeholders, combining lived experiences, operational insights, and functional ideas to design hybrid rooms that merge the advantages of both single- and multi-bed rooms. Technologies like BIM, VR, and game engines allowed stakeholders to view and evaluate the designs.

Our findings show the importance of flexibility in bedroom designs; hybrid rooms allow people who have had a stroke to adjust privacy and social interaction levels, aligning with the dynamic needs of stroke rehabilitation. Space and cost trade-offs are also important considerations. While hybrid designs like the *Butterfly* and *Quartet* require more spatial and initial financial investment than standard rooms, their perceived appropriateness for the stroke rehabilitation context may justify the expenditure.

The future of stroke rehabilitation room design should move beyond the single versus multi-bed debate and instead focus on adaptable, patient-centred solutions that balance privacy, social interaction, safety, and therapeutic functionality. Further research should specifically measure long-term clinical outcomes alongside economic impacts of hybrid rooms to inform evidence-based design guidelines. The focus of hospital bedroom design and any design evaluation should not be on the number of beds in the room, but rather, on whether the room meets the specific clinical and wellbeing needs of the users.

## Supporting information

S1 File Aggregated rankings data. (PDF)

S2 File Bedroom evaluations data. (PDF)

S3 File NOVELL VR and focus group questions. (PDF)
