# Supplementary material for: Ending the single versus multi-bedroom debate: Hybrid rooms meet stroke rehabilitation needs
Source: PLoS One. 2026 Sep 3;21(9):e0356623. doi: 10.1371/journal.pone.0356623 (PMC13541176; doi:10.1371/journal.pone.0356623)
Supplement: Supplementary file 1 [file pone.0356623.s001.pdf]

**Objective**	**Bedroom Design Alternativ**	**Condorset**	**Valency**	**ValTies**
Versatility	Quartet	4	4	1
Versatility	Butterfly	4	4	1
Versatility	Trokia	2	2	0
Versatility	Duet	1	1	0
Personal_control	Quartet	4	4	1
Personal_control	Butterfly	4	4	1
Personal_control	Trokia	2	2	0
Personal_control	Duet	1	1	0
Outdoor	Quartet	4	4	1
Outdoor	Butterfly	4	4	1
Outdoor	Trokia	2	2	0
Outdoor	Duet	1	1	0
Positive_stimulating	Quartet	4	4	1
Positive_stimulating	Butterfly	4	4	1
Positive_stimulating	Trokia	2	2	0
Positive_stimulating	Duet	1	1	0
Overall	Quartet	4	4	1
Overall	Butterfly	4	4	1
Overall	Trokia	2	2	0
Overall	Duet	1	1	0
