# Supplementary material for: Ending the single versus multi-bedroom debate: Hybrid rooms meet stroke rehabilitation needs
Source: PLoS One. 2026 Sep 3;21(9):e0356623. doi: 10.1371/journal.pone.0356623 (PMC13541176; doi:10.1371/journal.pone.0356623)
Supplement: Supplementary file 2 [file pone.0356623.s002.pdf]

Internal

Participant ID	versatility quartet	versatility duet	versatility butterfly	versatility troika	personal control quartet	personal control duet	personal control butterfly	personal control troika	outdoor and greenery quartet	outdoor and greenery duet	outdoor and greenery butterfly	outdoor and greenery troika	positive and stimulating quartet	positive and stimulating duet	positive and stimulating butterfly	positive and stimulating troika	overall quartet	overall duet	overall butterfly	overall troika
1	81.011986	17.760866	92.594109	51.486771	63.237087	10.51679	91.758598	53.647583	94.331436	7.763382	94.413254	66.458664	67.406601	10.379869	93.871536	61.361866	78.751045	30.134975	93.148354	57.10606
2	85.008743	16.687605	93.938354	8.823187	87.177742	6.157399	93.725082	5.118942	73.755547	6.507209	91.555389	6.117068	86.354561	2.951747	92.994278	19.264313	77.324173	16.426168	93.653336	12.676674
3	86.126778	55.198582	94.909821	71.08596	88.943695	34.496513	93.338257	69.730087	89.735962	55.232346	82.747047	81.540123	100	53.107716	84.702484	70.01535	93.025337	67.890182	90.384537	69.300079
4	76.897591	30.083385	88.959946	36.670959	74.297646	18.786661	90.463501	23.174961	71.468384	9.335552	94.416878	69.536087	72.93531	11.721781	94.706161	50	74.142334	14.086817	91.720398	68.279144
5	93.477837	6.836107	77.411224	24.257494	70.797554	31.808371	96.373001	22.970278	88.270592	2.178424	75.658104	70.513412	80.733276	0	68.948158	23.780123	98.10421	33.260979	89.304321	50
6	88.143921	30.226007	89.050804	64.691826	90.943398	14.053429	92.825478	65.505913	70.084015	20.605173	83.007935	72.554817	79.526527	25.058973	83.031433	67.325226	80.10453	26.152805	90.08519	77.193031
7	78.350334	61.84861	75.469101	61.815273	84.776337	63.464005	81.245445	57.698071	85.552628	59.363567	85.514465	59.943104	85.434555	62.90313	77.690819	61.931862	84.093903	65.583817	88.062553	65.027634
8	84.095604	50	81.736633	64.44883	84.343796	50	83.017609	66.59803	87.383804	15.818553	86.693748	84.849785	85.262535	17.609879	87.027184	63.741547	86.848404	50	87.000725	65.488686
9	67.946617	36.467453	69.87606	73.36306	68.599754	31.010544	70.732613	77.082649	76.646835	32.244923	79.689995	71.493881	83.904434	22.598516	72.215919	70.653008	81.499252	40.668968	70.92675	70.905075
10	77.00943	83.746788	82.438316	82.947105	82.339546	59.877373	90.403671	65.701256	61.490978	60.566353	96.78669	76.036598	64.051491	56.482208	91.697762	58.849068	75.016174	66.564148	91.069847	63.639938
11	87.085808	8.163336	71.802979	62.186794	72.444931	0	71.967522	55.488579	75.545486	2.282994	69.32383	60.733509	83.304642	2.649641	80.40506	50.560688	77.221207	13.808976	83.687485	63.298775
12	100	31.3242	91.928429	92.295715	100	34.548367	100	86.103111	100	67.785561	92.597229	92.919556	100	68.84124	93.417999	100	100	75.196869	92.770599	100
13	84.233063	42.072487	75.656502	50	93.846497	25.330257	81.301735	45.636452	97.820099	24.177221	92.344589	76.70462	89.173004	0	77.316727	57.387627	96.813538	19.475544	84.416481	50
14	77.496063	33.564575	96.737152	38.6479	84.600143	33.499931	83.913399	58.321232	86.671585	18.845995	86.675461	58.646797	82.103661	13.689772	90.229187	54.093689	82.47332	19.888977	89.974503	57.568645
15	82.323318	40.290001	89.493156	71.94809	80.817696	34.123657	84.973434	69.472763	83.722282	39.770809	85.75013	78.54454	84.341682	32.396561	84.956924	73.758827	82.952156	43.927967	89.66687	69.274391
16	76.337372	73.418236	96.842392	65.434578	90.401375	28.975567	97.723366	84.137047	82.817085	21.061853	91.22097	81.897263	99.996445	22.562357	100	73.62355	70.025513	25.951445	88.888481	80.030609
19	74.919556	11.415817	85.156326	69.178558	84.472343	36.347057	79.415108	69.582596	86.589622	23.755125	88.825836	78.465553	82.539856	7.17954	86.955833	65.490868	85.709343	16.951607	85.296295	79.223038
28	90.942169	2.957968	80.292114	11.793988	77.438011	0	100	8.774636	73.050491	0	78.729721	36.820065	76.338081	4.070377	88.08194	19.46554	67.985954	3.502197	52.747147	28.90324
29	50.349759	10.999994	73.301491	29.136253	54.517757	5.439192	50.485504	30.972811	54.133286	7.319822	60.024227	51.223492	55.303616	4.588784	50.389749	36.061836	63.737392	12.793471	70.215691	35.393551
30	84.771912	19.724014	85.220505	61.117767	85.595375	13.759618	78.689346	51.247169	89.54567	0	78.310471	71.26178	91.607773	6.812777	78.344704	55.50061	89.354385	6.419212	82.517609	56.596981
