# Supplementary material for: Ending the single versus multi-bedroom debate: Hybrid rooms meet stroke rehabilitation needs
Source: PLoS One. 2026 Sep 3;21(9):e0356623. doi: 10.1371/journal.pone.0356623 (PMC13541176; doi:10.1371/journal.pone.0356623)
Supplement: Supplementary file 3 [file pone.0356623.s003.pdf]

Internal

<u>Questions to prompt immediate feedback of the architectural designs.</u>
Participants will be encouraged to speak their thoughts throughout being in the VR as part of a 'talk aloud protocol' (e.g., participants might make statements such as: "I am seeing the nurses' station on my right, I like the way it looks but it feels a bit closed in."). Verbal responses will be audio-recorded.

Participants will be asked to answer some brief demographic questions, followed by a series of questions to gauge their feedback of the architectural design. Some questions will be presented as a Visual Analogue Scales (VAS) on an iPad, others will be presented as multiple choice and VAS questions presented within the virtual environment (see Figure 1).

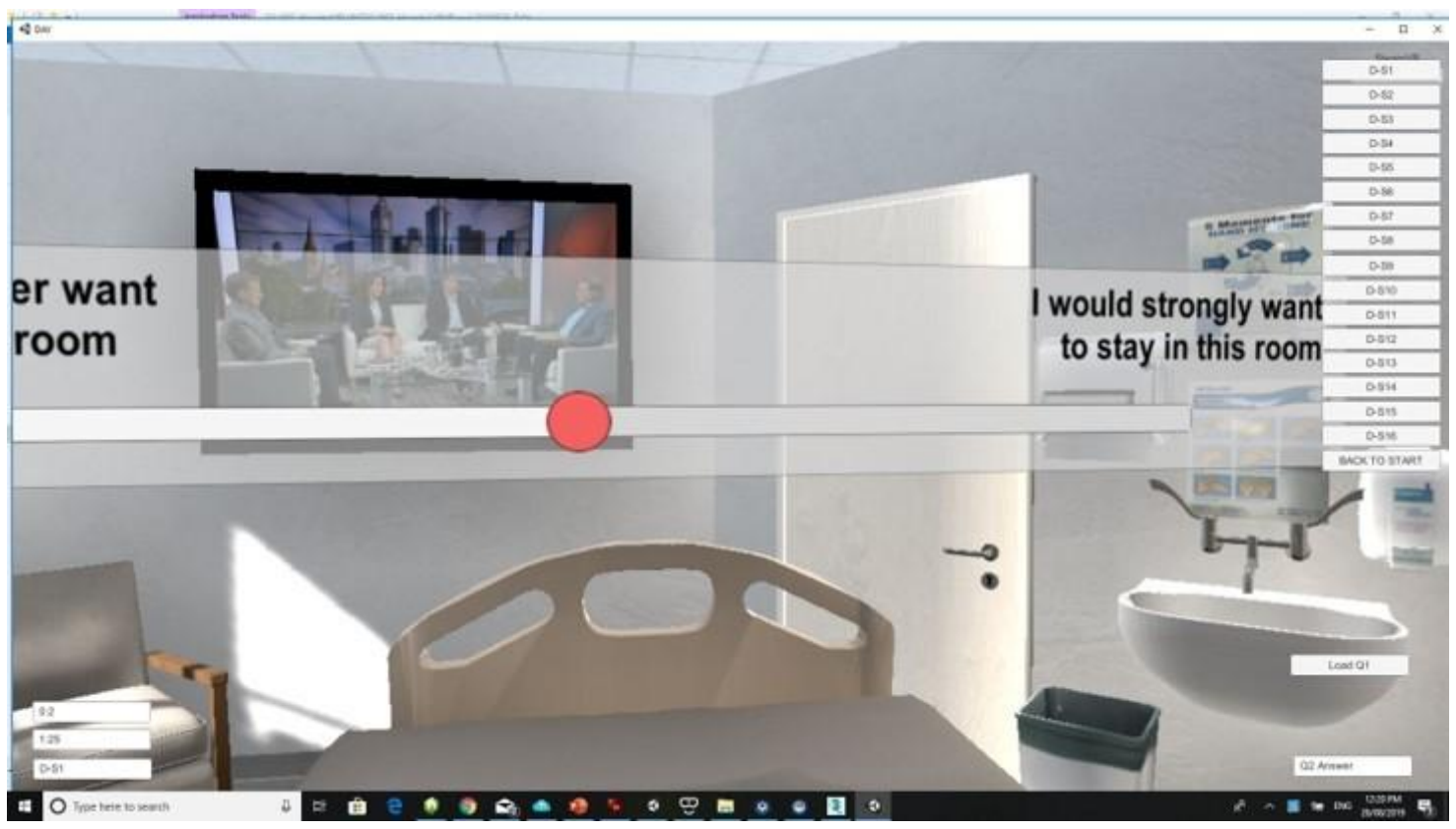

Figure 1. Example of a VAS presented within a virtual environment.

Each participant will be inducted to the VR session using the following briefing protocol:

> During the session you will be asked to answer some simple demographic questions before you will be asked to put on a high-end consumer grade head mounted virtual reality headset. In the VR environment, you will be shown a series of 3D rehabilitation wards and bedrooms. We will ask you to verbalise your experience (talk aloud about what you are seeing and your opinions) whilst in the VR environment which will be recorded with the use of an audio microphone, and what you see through the VR headset will be video captured.
>
> After you have experienced each VR scenario, you will be prompted to provide feedback about the space by asking a series of questions.
>
> Following the testing session, we will conduct a short focus group to ask you about your experiences of the virtual environments that you saw. This focus group will be audiorecorded.

Demographic questions
Which gender do you most identify with?
- Female
- Male
- Other

What is your age?
- 18-25 years
- 26-35 years
- 36-45 years
- 46-55 years
- 56-65 years
- 66+ years

Stroke survivors only: How many years are you post-stroke? ____ years

Have you used VR in the past?
- Many times
- Once or twice
- Never

If you used VR in the past, did you experience any motion sickness or other negative effects?
- Yes -> if participants answer 'yes' they will be asked not to participate
- No

Visual Analogue Scale (VAS) and multiple choice questions

1. Question 1 (First impressions):

"We're interested to hear about your first impressions of this [ward/bedroom]. Please give a short (30 second or less) summary of your initial thoughts."

"We would like to ask some more specific questions to understand your opinions about this ward/bedroom."

2. Question 2 (Versatility):

"Think about the versatility of this [ward/bedroom]. A space that is versatile and flexible can be adapted to meet the changing needs and preferences of stroke survivors. A versatile space promotes practice, activity, and rest; allowing stroke survivors to take appropriate risks based on their recovery level. Such a space can accommodate people with different abilities and promote efficiency and safety.

How versatile is this [ward/bedroom], on a scale from 0 to 100? Please indicate your response on the line below.

Where 0 = a ward/bedroom that is not at all versatile, and
100 = the most versatile ward/bedroom you can imagine."

3. Question 3 (personal control):

"Think about the extent to which stroke survivors have personal control over this [ward/bedroom]. If a space offers personal control, then stroke survivors should be able to make changes in the space themselves, or easily ask someone to help them do this. They should be able to add their own personal

touches to a space, and to adjust the space to be more private or social depending on their needs and preferences. Having personal control over a space can remind a person that their feelings and preferences are respected, help them feel a sense of ownership and agency, and improve emotional well-being.

How much personal control does this [ward/bedroom] provide to stroke survivors, on a scale from 0 to 100? Please indicate your response on the line below.

Where 0 = a ward/bedroom that provides no personal control, and
100 = a ward/bedroom that provides the most personal control that you can imagine."

4. Question 4 (outdoor and green spaces):

"Think about access to the outdoors, nature, and green spaces from this [ward/bedroom]. A ward/bedroom with very good access would have an easily accessible door to the outside and nature, but also views of the outside and nature. The outdoor environment should make you feel good. Access to the outdoors can help to promote activity, is important for connection to the outside community and the real world, and beneficial for emotional well-being.

How good is the access to the outdoors and nature in this [ward/bedroom], on a scale from 0 to 100? Please indicate your response on the line below.

Where 0 = a ward/bedroom that provides no access to the outside or nature, and
100 = a ward/bedroom that provides the best access to the outside or nature that you can imagine."

5. Question 5 (positive and stimulating):

"Think about whether this [ward/bedroom] feels positive, stimulating, and fun in a way that would promote activity and emotional well-being for stroke survivors. Such spaces should have engaging, uplifting, attractive, and interesting destinations both indoors and outdoors, to draw people out of their beds. It's important to have a variety of options that cater to different interests and tastes, with new things on offer regularly to promote continued engagement.

How positive and stimulating is this [ward/bedroom], on a scale from 0 to 100? Please indicate your response on the line below.

Where 0 = a ward/bedroom that is not at all positive or simulating, and
100 = the most positive and stimulating ward/bedroom that that you can imagine."

6. Question 6 (Legibility) [WARD designs only]:

"Think about whether this ward feels legible to you. A legible space is easy to navigate and understand, and users should be able to intuitively find key destinations. This requires not just clear signage, but also

environmental cues. A legible space should be accessible for all users, including those with low literacy, language barriers, or sensory impairments. A legible space can help promote a sense of belonging and reduce anxiety for stroke survivors, promote efficiency for staff, and increase safety for all users.

How legible is this ward, on a scale from 0 to 100? Please indicate your response on the line below.

Where 0 = a ward that is not at all legible (you feel lost all the time and don't know where anything is), and
100 = the most legible ward that that you can imagine (you easily know where everything is and how to get there)."

7. Question 7 (Overall):
"Overall, the best possible [ward/bedroom] for stroke rehabilitation should do the following: 1) help to optimise clinical outcomes by promoting activity (physical, cognitive, social) and rest, 2) operate efficiently, 3) maximise the emotional well-being of all users, and 4) keep all users safe.

Overall, how well do you think this [ward/bedroom] achieves these things, on a scale from 0 to 100?

Where 0 = the worst ward/bedroom that you can imagine, and
100 = the best ward/bedroom that you can imagine?"

8. Question 8 (Open ended):
"Please tell us more about your response to the previous question. What was it about this ward/bedroom that motivated your response?"

The following questions will be asked in some of the bedroom designs only:

9. "How do you feel in the room?"

a) Calm
b) Relaxed
c) Cheerful
d) Excited
e) Sad
f) Bored
g) Tense
h) Irritated

10. “How much do you want to stay in the room?” (on a sliding scale, from 0 to 100)

Where 0 = I don't want to stay in the room, and

100 = I strongly want to stay in the room

11. “How much does the view make you want to get out of bed?” (on a sliding scale, from 0 to 100)

Where 0 = I don’t want to get out bed at all, and

100 = I very much want to get out of bed

12. “If you get out the bed, where do you want to go?”

i) Window seat
j) Outside
k) Corridor

The following questions will be asked in some of the corridor designs only:

13. “Did you notice the gym area in this scenario?”

Yes/No

14. “Did you notice the communal area in this scenario?”

Yes/No

15. “How much did you want to go to the gym in this scenario?” (on a sliding scale, from 0 to 100)

Where 0 = Do not want to go to the gym at all, and

100 = extremely want to go to the gym.

16. “What most influenced your response to the previous question?”

a) Distance to the gym
b) Being able to see the gym
c) The look of the gym

17. “How much did you want to go to the communal area in this scenario?” (on a sliding scale, from 0 to 100)

Where 0 = Do not want to go to the communal area at all, and

100 = extremely want to go to the communal area.

18. “What most influenced your response to the previous question?”

a) Distance to the communal area
b) Being able to see the communal area
c) The look of the communal area

19. “In your opinion, was the distance of the gym in this scenario?”

l) Too close
m) Just right
n) Too far

20. “In your opinion, was the distance of the communal area in this scenario?”

a) Too close
b) Just right
c) Too far

Focus group questions

After viewing the architectural designs in VR, and answering the associated VAS questions, participants will join a focus group session to gain more detailed information about which of the alternatives they preferred, and why, i.e., what motivated their responses to the VAS questions.

To prompt recall of the different alternatives, participants will be shown screenshots of the designs. These visual prompts will remain visible throughout the focus group.

The following questions will be used as prompts to guide discussion during the focus group. We will follow a semi-structured question format to explore participants’ opinions fully, whereby additional questions (not listed below) may be asked to probe further into participants’ responses.

1. “Which ward/bedroom did you think would best help to promote activity/encourage people to leave their bed? And why?”

2. “Which ward/bedroom did you think would best help to promote rest? And why?”
3. “Which ward/bedroom did you think would best help to promote emotional well-being? And why?”
4. “Which ward/bedroom did you think would be the safest? And why?”
5. “Which ward/bedroom did you think would operate the most efficiently? And why?”
6. “Which was your preferred ward/bedroom? And why?”
7. “After participating in this focus group, if you were to view the alternatives in VR again, would you answer the VAS questions differently? Why? Or in other words, has your opinion about the alternatives/preferences for the different alternatives changes after doing the focus group?”
