# Supplementary material for: Ending the single versus multi-bedroom debate: Hybrid rooms meet stroke rehabilitation needs
Source: PLoS One. 2026 Sep 3;21(9):e0356623. doi: 10.1371/journal.pone.0356623 (PMC13541176; doi:10.1371/journal.pone.0356623)
Supplement: Supplementary file 5 [file pone.0356623.s005.docx]

**Responses to Academic Editor and Reviewers**	23 April 2026

PONE-D-25-63722

Ending the Single versus Multi-Bedroom Debate: Hybrid rooms meet stroke rehabilitation needs

PLOS One

1. Please ensure that your manuscript meets PLOS ONE's style requirements, including those for file naming. The PLOS ONE style templates can be found at

<https://journals.plos.org/plosone/s/file?id=wjVg/PLOSOne_formatting_sample_main_body.pdf> and

<https://journals.plos.org/plosone/s/file?id=ba62/PLOSOne_formatting_sample_title_authors_affiliations.pdf>

Agree: We have updated the document to PLOS ONE’s style template.

2. Thank you for stating the following in the Acknowledgments Section of your manuscript: “We would like to thank The Florey Institute of Neuroscience, The Felton Bequest and The University of Melbourne for generously funding this project.” We note that you have provided funding information that is not currently declared in your Funding Statement. However, funding information should not appear in the Acknowledgments section or other areas of your manuscript. We will only publish funding information present in the Funding Statement section of the online submission form. Please remove any funding-related text from the manuscript and let us know how you would like to update your Funding Statement. Currently, your Funding Statement reads as follows:  “The Florey Institute of Neuroscience, The Felton Bequest and The University of Melbourne” Please include your amended statements within your cover letter; we will change the online submission form on your behalf.

Agree: The Funding information has been removed from the text in the manuscript. The funding parties for the project are: The Felton Bequest and The University of Melbourne. In addition, Julie Bernhardt was funded by an NHMRC Research Fellowship (1154904). The Florey Institute of Neuroscience and Mental Health acknowledges support from the Victorian government and in particular funding from the Operational Infrastructure Support Grant. We have included our amended statements within the cover letter above. Thank you in advance for changing the online submission form on our behalf.

3. We note that the grant information you provided in the ‘Funding Information’ and ‘Financial Disclosure’ sections do not match.  When you resubmit, please ensure that you provide the correct grant numbers for the awards you received for your study in the ‘Funding Information’ section.

Agree: The Felton Bequest grant and University of Melbourne matched funding did not have grant numbers. We have provided the NHMRC Research Fellowship Number for Julie Bernhardt.

4. Thank you for stating the following financial disclosure: “The Florey Institute of Neuroscience, The Felton Bequest and The University of Melbourne” Please state what role the funders took in the study.  If the funders had no role, please state: "The funders had no role in study design, data collection and analysis, decision to publish, or preparation of the manuscript." If this statement is not correct you must amend it as needed.  Please include this amended Role of Funder statement in your cover letter; we will change the online submission form on your behalf.

Note: The statement is correct.

5. We note that your Data Availability Statement is currently as follows: “All relevant data are within the manuscript and its Supporting Information files. Please confirm at this time whether or not your submission contains all raw data required to replicate the results of your study.

Note: We selected the wrong option for data disclosure. Data cannot be shared publicly because of limited consent provided per the ethics requirements. Data are available from the Institutional Data Access / Ethics Committee. The ethics committee contact details are as follows: Tel (03) 9214 3845 or +61 3 9214 3845 or [resethics@swin.edu.au](mailto:resethcs@swin.edu.au) Swinburne University Technology Human Research Ethics Committee (reference: 20204154-4993, 20234154-15784): The Neuroscience Optimised Virtual Environment Living Lab (NOVELL) project: The NOVELL Redesign workshops.

6. One of the noted authors is a group or consortium “NOVELL Redesign”. In addition to naming the author group, please list the individual authors and affiliations within this group in the acknowledgments section of your *manuscript*. Please also indicate clearly a lead author for this group along with a contact email address.

The NOVELL Redesign Collaboration includes over 130 people. We have created a supplementary file listing all members of the NOVELL Redesign Collaboration as a hyperlink and have referenced this supplementary file within the manuscript Acknowledgement Section of the paper.

7. Your ethics statement should only appear in the Methods section of your manuscript. If your ethics statement is written in any section besides the Methods, please move it to the Methods section and delete it from any other section. Please ensure that your ethics statement is included in your manuscript, as the ethics statement entered into the online submission form will not be published alongside your manuscript.

Noted. We have moved the ethics statement to the Methods section and deleted it from all other sections.

8. We note that Figures 2, 8, 9, 10 and 11 in your submission contain copyrighted images. All PLOS content is published under the Creative Commons Attribution License (CC BY 4.0), which means that the manuscript, images, and Supporting Information files will be freely available online, and any third party is permitted to access, download, copy, distribute, and use these materials in any way, even commercially, with proper attribution. For more information, see our copyright guidelines: <http://journals.plos.org/plosone/s/licenses-and-copyright>.

Note: The images in Figures 2, 8, 9, 10, and 11 were created by us, the authors. We provide permission for these to be published and reused with appropriate attribution.

9. We note that Figures 3, 4, 5 and 10 includes an image of a [patient / participant / in the study].

As per the PLOS ONE policy (<http://journals.plos.org/plosone/s/submission-guidelines#loc-human-subjects-research>) on papers that include identifying, or potentially identifying, information, the individual(s) or parent(s)/guardian(s) must be informed of the terms of the PLOS open-access (CC-BY) license and provide specific permission for publication of these details under the terms of this license. Please download the Consent Form for Publication in a PLOS Journal (<http://journals.plos.org/plosone/s/file?id=8ce6/plos-consent-form-english.pdf>). The signed consent form should not be submitted with the manuscript, but should be securely filed in the individual's case notes. Please amend the methods section and ethics statement of the manuscript to explicitly state that the patient/participant has provided consent for publication: “The individual in this manuscript has given written informed consent (as outlined in PLOS consent form) to publish these case details”.

If you are unable to obtain consent from the subject of the photograph, you will need to remove the figure and any other textual identifying information or case descriptions for this individual.

Note: Figure 3 includes the faces of one of the authors (Prof Marcus White) and one of the participants. This participant has been asked for her permission and has signed the Consent Form for Publication in a PLOS Journal. We have stored this signed consent form securely. The third person in this figure is not identifiable (face obscured).

Figure 4 does not include any identifiable figures, only hands/back of head. Figure 5 – the sections of the figure that included people have been blurred to obscure identity. (Please see updated Fig 5 R1)

Figure 10 includes only animated figures (not real people).

10. We note that this data set consists of interview transcripts. Can you please confirm that all participants gave consent for interview transcript to be published?

Note: Participants did not provide consent for these transcripts to be published. Deidentified excerpts from the focus groups (quotes, sections) have been included in supplementary material. The full audio recordings and transcripts will be retained for 5 years post publication, as per ethical approval from the Swinburne University Human Ethics committee). Swinburne University Technology Human Research Ethics Committee (reference: 20204154-4993, 20234154-15784): The Neuroscience Optimised Virtual Environment Living Lab (NOVELL) project: The NOVELL Redesign workshops.

Researchers who meet the criteria for accessing confidential data may access the dataset by contacting the ethics committee contact details are as follows: Tel (03) 9214 3845 or +61 3 9214 3845 or [resethics@swin.edu.au](mailto:resethcs@swin.edu.au)

11. If the reviewer comments include a recommendation to cite specific previously published works, please review and evaluate these publications to determine whether they are relevant and should be cited. There is no requirement to cite these works unless the editor has indicated otherwise.

Noted with thanks.

# Responses to Reviewer #1:

1. The paper, though conceptually interesting, lacks a clear hypothesis and defined research gap. The “hybrid room” concept is presented as an outcome rather than a solution derived from a well-structured problem statement. Authors should clarify the **specific research question driving** the study and how it advances beyond previous work on stroke-rehabilitation environments and evidence-based healthcare design???

Agree: We have clarified specific research questions (added to para 2, page 6). We have also elaborated on the advances to previous work on rehabilitation and EBD in the Discussion section (para 1, page 24; para 5, Page 25).

2. While the “Provotype Esquissé Workshops” and VR simulations are detailed in narrative form, the analytical rigor is missing. The manuscript must specify the quantitative framework, e.g., …sample size justification, statistical validation of responses, reliability measures, and criteria used for performance comparison between room types.

Agree: We have added additional detail in the method section of the paper to emphasise that this study was an early pilot, or proof-of-concept to explore whether the VR evaluation approach could be effectively integrated in the co-design process to allow user experts to provide input and evaluation of emerging design options.

Further emphasis has also been placed in the Methods on the predefined criteria (key objectives) that participants used to evaluate the four design options. These predefined criteria are reflected in the design priorities, the VAS evaluation questions, and the focus group questions.

The intention of the qualitative and quantitative measures in this study was to a) explore whether the VR evaluation approach resulted in observable differences in preferences from the participants for the different bedroom designs – Can users perceive differences and report preferences between design options when they are displayed in VR? And b) begin to explore which of the bedroom options were preferred and why. To address point b, we did not intend to statistically compare between the bedroom design options, rather we asked participants to rank how well each of the bedroom options performed according to each of the pre-defined criteria, and we combined these rankings using a graph-theory-based algorithm to determine an aggregated ranking of the bedrooms for each of the predefined objectives.

This has been clarified in the Methods (para 1, 3 and 4, page 9; para 3, 4, page 10) and is reflected in new sections in the Results (page 20).

3. The paper mentions 20 participants but gives no information on inclusion criteria, demographic distribution, prior VR experience, or potential bias introduced by training or familiarity with design software. The authors should demonstrate whether the sample size is statistically adequate for deriving meaningful conclusions.

Agree: Further information has been added to the Method regarding inclusion criteria (page 9) and demographic summaries have been added to the Results (page 20). An explanation regarding sample size has been added to the Methods (page 7) - the sample was not determined a priori, as no statistical comparisons were planned, rather the emphasis was on achieving representation across each of the key expert stakeholder groups.

4. Graphical data (e.g., Figure 12) are mentioned but not accompanied by statistical indicators such as confidence intervals, p-values, or effect sizes. Without these, the claimed superiority of the “Butterfly” or “Quartet” designs remains subjective.

Agree: We have added to the Results section to detail the results of our ranking analysis (see page 21).

We have also adjusted the tone of the reporting of findings throughout the manuscript (examples: para 2, 3, p.2 ; para 4, p2 ; and para 1, 2 p.27).

5. The terminology “hybrid rooms” and “provotypes” needs rigorous definition and comparison with existing healthcare architectural typologies. It is unclear how these differ structurally or functionally from established design principles or prior adaptive modular systems used in stroke rehabilitation units.

Agree We have elaborated on the definitions and further explained from where the ’typical’ two bedroom layout came (para. 2, p.8, and para. 3 & 4, p.15). Previous reviews of existing building typologies for stroke rehabilitation suggest that there are very few adaptive modular systems used in stroke rehabilitation units (Described in Yang et al.’s desktop review), we hope that our study and the early support of the hybrid design options afforded by our participants will begin to rectify this.

6. The study would be strengthened by referencing or aligning findings with measurable rehabilitation indicators, mobility improvement, patient satisfaction, safety metrics, or recovery duration.

Agree: The key objectives for optimal stroke rehabilitation facility design that were used to inform the bedroom designs and evaluation criteria in this study (see page 11) have been clearly linked in previous publications with measurable rehabilitation indicators including physical, cognitive, and social activity (which is essential for brain repair and re-learning of skills or abilities impacted by stroke), the safety and emotional wellbeing of the person who has had a stroke, and the efficiency of the clinical care. We agree with the reviewer that we had not emphasised this connection clearly enough in the manuscript. We have added sentences to the Introduction to clarify this (page 6). We have also added sentences to the Discussion (page 24) to suggest that, while this study did not look at clinical outcomes, future research could investigate the connection between the presence of desired design features in existing stroke rehabilitation facilities and the presence of behaviours that are beneficial for recovery (e.g., physical and social activity).

7. Statements about long-term cost efficiency, patient satisfaction, and improved outcomes are speculative without quantitative or longitudinal evidence. Authors should carefully delimit the claims to what their data can substantiate.

Agree: We have clarified that the speculative nature of cost efficiency/outcomes statements (in this paper), and point to concurrent research that provides more detailed short and long-term cost benefit findings (Limitations and suggested further research, Para. 1, 4, p.27).

8. The literature review, though broad, does not sufficiently integrate recent systematic evidence from neuro-architecture and rehabilitation design studies.

Agree: We have updated the literature review to integrate recent neuro-architecture and rehabilitation design studies (Para. 2, 3, 4, page.4)

9. Ethical approval and participant consent are briefly mentioned but lack details about data handling, anonymization, and reproducibility of the digital VR environments, which is crucial for research transparency under PLOS ONE standards.

Agree: We have added details of data handling and anonymisation (para.4, p.9) as well as further clarification on digital VR environments (para. 4, 5, pape.7).

10. Finally, the language and tone of the manuscript are sometimes promotional and design-studio-oriented rather than scientific…

Agree: As mentioned above, we have adjusted tone across the paper.

# Responses to Reviewer #2:

Thank you for submitting your manuscript to this journal. This manuscript presents an innovative and interdisciplinary study addressing the longstanding single versus multi-bed debate in stroke rehabilitation environments. The integration of speculative design workshops, BIM modelling, and immersive VR testing represents a strong methodological contribution. The study is clearly written, logically structured, and grounded in prior work on value-focused design and rehabilitation architecture. However, several methodological and analytical concerns limit the strength of the conclusions in their current form. The following are my comments about this manuscript.

1) First, the study includes only 20 participants in the VR evaluation phase. While purposive sampling of experts and stroke survivors is reasonable for exploratory design research, the manuscript makes comparative claims regarding the superiority of specific room types. Without justification of sample adequacy or power considerations, these claims appear stronger than the data can support.

Agree: We have added additional detail in the method section of the paper to outline the quantitative framework and explanation of sample size (para 1, 3, p.9). We agree that some of our claims were not well-supported by the data. We have adjusted the tone of our claims in multiple areas (para 2, 3, p.2 ; para 4, p2 ; and para 1, 2 p.24).

2) Second, the statistical analysis is limited to descriptive statistics. Median and interquartile range are reported, but no inferential tests were performed to examine differences between room types. Given that participants evaluated all four designs, a non-parametric repeated-measures analysis such as a Friedman test would have been appropriate. Post hoc comparisons could further clarify whether observed differences are statistically meaningful rather than impressionistic.

Agree: Detail was lacking in the Methods and Results to describe the extent of our analysis. We did not intend to statistically compare between the bedroom design options, rather we asked participants to rank how well each of the bedroom options performed according to each of the pre-defined criteria, and we combined these rankings using a graph-theory-based algorithm to determine an aggregated ranking of the bedrooms for each of the predefined objectives.

This has been clarified in the Methods (page 10) and is reflected in new sections in the Results (page20, 21, 22).

3) Third, the Visual Analogue Scale instrument appears to have been adapted for this study, yet no information is provided regarding reliability, internal consistency, or construct validity. The manuscript would benefit from reporting psychometric properties or at least acknowledging the exploratory nature of these measures.

Agree: We have relevant citations of previous studies that confirm the effectiveness of the VAS within VR in Stroke Rehabilitation contexts (Para.4, p.10). The VAS questions are not a psychometric instrument, rather they were chosen as an accessible, quick, easy to comprehend question format that could be presented within the VR environment. In addition, VAS responses yield continuous data (scale from 0 to 100) which are ideal for subsequently ranking the bedroom options. The constructs that were measured by the VAS (versatility, personal control, outdoor and green spaces, and positive and stimulating environment) have been well-established as critical criteria for stroke rehabilitation environment design and were predefined as the key criteria for design and evaluation in this study.

4) Fourth, participant recruitment relied on existing collaborative networks. While understandable, this introduces potential bias, including familiarity bias and design-team proximity bias. The manuscript should discuss this more explicitly as a limitation.

Agree: We have added clarification on the potential biases in the Discussion section of the paper (para.4 p.27).

5) Fifth, several claims in the discussion extend beyond the measured outcomes. The study evaluates perceived suitability and stakeholder preference within VR environments. It does not measure clinical recovery, healthcare cost reduction, or long-term functional outcomes. Statements suggesting improved recovery efficiency, reduced long-term disability, or cost savings should be reframed as hypotheses rather than conclusions.

Agree: We had adjusted the discussion and conclusion sections to reflect the nature of the results. We have also adjusted tone in multiple areas (para 1, 2, 3, p24; para 4-5 p25; para 2, p26; para 1, 2 and 4, p.27 and para 6, p.28).
