# Supplementary material for: Ending the single versus multi-bedroom debate: Hybrid rooms meet stroke rehabilitation needs
Source: PLoS One. 2026 Sep 3;21(9):e0356623. doi: 10.1371/journal.pone.0356623 (PMC13541176; doi:10.1371/journal.pone.0356623)
Supplement: Supplementary file 6 [file pone.0356623.s006.docx]

**PONE-D-25-63722R1**

Ending the Single versus Multi-Bedroom Debate: Hybrid rooms meet stroke rehabilitation needs

**Responses to Academic Editor and Reviewers**

**Comments to the Author**

1. If the authors have adequately addressed your comments raised in a previous round of review and you feel that this manuscript is now acceptable for publication, you may indicate that here to bypass the “Comments to the Author” section, enter your conflict of interest statement in the “Confidential to Editor” section, and submit your "Accept" recommendation.

Reviewer #1: All comments have been addressed

Reviewer #2: All comments have been addressed

2. Is the manuscript technically sound, and do the data support the conclusions?

The manuscript must describe a technically sound piece of scientific research with data that supports the conclusions. Experiments must have been conducted rigorously, with appropriate controls, replication, and sample sizes. The conclusions must be drawn appropriately based on the data presented.

Reviewer #1: Yes

Reviewer #2: Yes

3. Has the statistical analysis been performed appropriately and rigorously?

Reviewer #1: Yes

Reviewer #2: N/A

4. Have the authors made all data underlying the findings in their manuscript fully available?

The [PLOS Data policy](http://track.editorialmanager.com/CL0/http:%2F%2Fwww.plosone.org%2Fstatic%2Fpolicies.action%23sharing/1/010f019e2522fe6a-170ad329-a288-48fb-8e9d-fe60508e331f-000000/x1Ys9_7AxHjydq2OltjzZzHZVHU-4TWk7jqwhRAXNeU=258) requires authors to make all data underlying the findings described in their manuscript fully available without restriction, with rare exception (please refer to the Data Availability Statement in the manuscript PDF file). The data should be provided as part of the manuscript or its supporting information, or deposited to a public repository. For example, in addition to summary statistics, the data points behind means, medians and variance measures should be available. If there are restrictions on publicly sharing data—e.g. participant privacy or use of data from a third party—those must be specified.

Reviewer #1: Yes

Reviewer #2: Yes

5. Is the manuscript presented in an intelligible fashion and written in standard English?

PLOS ONE does not copyedit accepted manuscripts, so the language in submitted articles must be clear, correct, and unambiguous. Any typographical or grammatical errors should be corrected at revision, so please note any specific errors here.

Reviewer #1: Yes

Reviewer #2: Yes

**6. Review Comments to the Author**

Reviewer #1: Revised version has been improved and is recommended for the publication. Great efforts by the authors.

Reviewer #2: Thank you for the comprehensive revision. The manuscript has improved substantially, and the methodological transparency is now much clearer. The study is technically sound within its stated exploratory and proof-of-concept scope. The justification of sample size, clarification of recruitment strategy, and more cautious framing of conclusions have strengthened the manuscript considerably.

The use of a values-based design brief, combined with immersive VR evaluation and multi-stakeholder input, represents a meaningful contribution to healthcare architecture and evidence-based design research. The data presented support the conclusions as framed, particularly because the study positions itself as exploratory and preference-based rather than confirmatory or outcome-driven. Specifically, I suggest:

1. Slightly tempering any remaining language in the abstract and conclusion that may imply direct improvement in clinical outcomes, clarifying that these findings indicate perceived suitability and stakeholder preference rather than measured rehabilitation effects.

Agree: We have edited the abstract and conclusions accordingly.

The abstract (p2) now reads:

*Stroke is a leading cause of disability worldwide, and stroke rehabilitation plays a critical role in recovery. Early rehabilitation often occurs in a hospital setting, but the hospital built environment is rarely designed to support the specific needs and priorities of people who have had a stroke. In recent decades, there has been an ongoing debate regarding the benefits of single versus multi-bed rooms in healthcare settings. This debate centres around balancing privacy and rest, with social interaction and safety. While single rooms may be beneficial for some people, multi-bed rooms may be better for others. Both options, however, come with their own set of drawbacks in the context of stroke rehabilitation.*

*In this study, we conducted a collaborative Living Lab to design a series of provocative-prototype hybrid room typologies that merged different aspects of single and multi-bed rooms to provide options specific for stroke rehabilitation. These typologies were used to generate four alternative bedroom designs which were modelled in virtual reality. The designs were informed by a values-based brief, outlining a set of predefined key criteria for optimising stroke rehabilitation environments: opportunities for activity and rest, wellbeing, safety, efficiency, flexibility, personal control, access to outdoors and green space, and access to a positive and stimulating environment. Twenty lived-experience and professional experts evaluated these virtual designs against the predefined values-based criteria.*

*Evaluation revealed two preferred hybrid hospital room designs that incorporated beneficial features of both multi-bed and single-bed rooms. Our approach of values-based design and evaluation within a virtual environment proved an effective means of generating and evaluating design options with meaningful input from a wide range of stakeholders. These findings highlight the potential for flexible and adaptable room design to meet the specific needs and priorities of people participating in early-stage rehabilitation after stroke.*

The Conclusion (p24) now reads:

*Our findings demonstrate the potential for using hybrid rooms to achieve the best of both Single and Multi-Bedroom types in a hospital setting. To address the Single vs. Multi-Bedroom debate in stroke rehabilitation, we co-designed and evaluated hypothetical hybrid room designs aimed at meeting objectives identified by stakeholders. We integrated input from multiple stakeholders, combining lived experiences, operational insights, and functional ideas to design hybrid rooms that merge the advantages of both single- and multi-bed rooms. Technologies like BIM, VR, and game engines allowed stakeholders to view and evaluate the designs.*

*Our findings show the importance of flexibility in bedroom designs; hybrid rooms allow people who have had a stroke to adjust privacy and social interaction levels, aligning with the dynamic needs of stroke rehabilitation. Space and cost trade-offs are also important considerations. While hybrid designs like the Butterfly and Quartet require more spatial and initial financial investment than standard rooms, their perceived appropriateness for the stroke rehabilitation context may justify the expenditure.*

*The future of stroke rehabilitation room design should move beyond the single versus multi-bed debate and instead focus on adaptable, patient-centered solutions that balance privacy, social interaction, safety, and therapeutic functionality. Further research should explore the long-term clinical and economic impacts of hybrid rooms to inform evidence-based design guidelines. The focus of hospital bedroom design and any design evaluation should not be on the number of beds in the room, but rather, on whether the room meets the specific clinical and wellbeing needs of the users.*

2. Briefly expanding the discussion of potential bias due to prior participant involvement in the NOVELL project.

Agree: We have elaborated on this further in the Limitations (pg. 23). In addition, to check for familiarity bias, we have calculated the aggregated rankings for two subgroups: 1) participants who had participated in previous NOVELL workshops (non-novices) and 2) participants who were novice to the project. Reassuringly, we have found that the butterfly and quartet designs have remained in the top two rankings, and the duet has the lowest ranking, for all the predefined objectives for both of these subgroups. We have added this to the Methods (pg. 11), Results (pg. 18) and Limitations (pg. 23). This section of the Limitations now reads:

*While we included a mix of participants familiar with the project and those who were unfamiliar with the project (novices), it should be noted that participant recruitment relied on existing collaborative networks. None of the participants who were familiar with the NOVELL project (non-novices) had seen the final provotypes prior to evaluating them in VR, but some were involved in the initial speculative design esquisses. The network-based recruitment and involvement in earlier stages of the project introduces potential bias, including research-team proximity and familiarity bias which may skew results in favor of designs or concepts that participants were already familiar with. Participants did not, however, favor the duet design, despite this design arguably being the most familiar as it drew heavily from currently Australian guidelines and norms. Reassuringly, we did not observe any notable differences in responses between participants who were familiar with the project (non-novices) versus those who were unfamiliar (novices); aggregated rankings of the bedroom provotypes for both subgroups placed the butterfly and quartet provotypes in the top two. In future expansion of this research, however, wider reaching recruitment methods should be included to ensure a wide range of opinions, including people who are not familiar with the priorities and objectives that were defined in the NOVELL project.*

3. Adding one clarifying sentence regarding the robustness of the ranking aggregation method to enhance transparency.

Agree: We thank the reviewer for this recommendation. A sentence has been added to the Methods (pg. 11) to describe the robustness of the aggregation algorithm compared to other methods for combining rankings: “*This aggregation method is a robust way of combining participant’s rankings because it treats rankings as purely ordinal data and uses graph theory to extract only the ordering that the data genuinely support—i.e., allowing ties between the provotypes—rather than summing ranks or forcing a definitive ranking [42].*”

These are minor clarifications rather than substantive changes.
