# Supplementary material for: Ending the single versus multi-bedroom debate: Hybrid rooms meet stroke rehabilitation needs
Source: PLoS One. 2026 Sep 3;21(9):e0356623. doi: 10.1371/journal.pone.0356623 (PMC13541176; doi:10.1371/journal.pone.0356623)
Supplement: Peer review history

# Peer review: Decision Letter 0

**DOI:** 10.1371/journal.pone.0356623.r001

**Authors:** Ruixin Liang

27 Feb 2026

Dear Dr. Lam,

Thank you for submitting your manuscript to PLOS ONE. After careful consideration, we feel that it has merit but does not fully meet PLOS ONE’s publication criteria as it currently stands. Therefore, we invite you to submit a revised version of the manuscript that addresses the points raised during the review process.

Please submit your revised manuscript by Apr 13 2026 11:59PM. If you will need more time than this to complete your revisions, please reply to this message or contact the journal office at plosone@plos.org. When you're ready to submit your revision, log on to https://www.editorialmanager.com/pone/ and select the 'Submissions Needing Revision' folder to locate your manuscript file.

- A letter that responds to each point raised by the academic editor and reviewer(s). You should upload this letter as a separate file labeled 'Response to Reviewers'.
- A marked-up copy of your manuscript that highlights changes made to the original version. You should upload this as a separate file labeled 'Revised Manuscript with Track Changes'.
- An unmarked version of your revised paper without tracked changes. You should upload this as a separate file labeled 'Manuscript'.

If you would like to make changes to your financial disclosure, please include your updated statement in your cover letter. Guidelines for resubmitting your figure files are available below the reviewer comments at the end of this letter.

If applicable, we recommend that you deposit your laboratory protocols in protocols.io to enhance the reproducibility of your results. Protocols.io assigns your protocol its own identifier (DOI) so that it can be cited independently in the future. For instructions see: https://journals.plos.org/plosone/s/submission-guidelines#loc-laboratory-protocols. Additionally, PLOS ONE offers an option for publishing peer-reviewed Lab Protocol articles, which describe protocols hosted on protocols.io. Read more information on sharing protocols at https://plos.org/protocols?utm_medium=editorial-email&utm_source=authorletters&utm_campaign=protocols.

We look forward to receiving your revised manuscript.

Kind regards,

Dr. Ruixin Liang

Guest Editor

PLOS One

Journal Requirements:

When submitting your revision, we need you to address these additional requirements.

1. Please ensure that your manuscript meets PLOS ONE's style requirements, including those for file naming. The PLOS ONE style templates can be found at

https://journals.plos.org/plosone/s/file?id=wjVg/PLOSOne_formatting_sample_main_body.pdf and

https://journals.plos.org/plosone/s/file?id=ba62/PLOSOne_formatting_sample_title_authors_affiliations.pdf

2. Thank you for stating the following in the Acknowledgments Section of your manuscript:

“We would like to thank The Florey Institute of Neuroscience, The Felton Bequest and The University of Melbourne for generously funding this project.”

We note that you have provided funding information that is not currently declared in your Funding Statement. However, funding information should not appear in the Acknowledgments section or other areas of your manuscript. We will only publish funding information present in the Funding Statement section of the online submission form.

Please remove any funding-related text from the manuscript and let us know how you would like to update your Funding Statement. Currently, your Funding Statement reads as follows:

“The Florey Institute of Neuroscience, The Felton Bequest and The University of Melbourne”

Please include your amended statements within your cover letter; we will change the online submission form on your behalf.

3. We note that the grant information you provided in the ‘Funding Information’ and ‘Financial Disclosure’ sections do not match.

When you resubmit, please ensure that you provide the correct grant numbers for the awards you received for your study in the ‘Funding Information’ section.

4. Thank you for stating the following financial disclosure:

“The Florey Institute of Neuroscience, The Felton Bequest and The University of Melbourne”

Please state what role the funders took in the study.  If the funders had no role, please state: "The funders had no role in study design, data collection and analysis, decision to publish, or preparation of the manuscript."

If this statement is not correct you must amend it as needed.

Please include this amended Role of Funder statement in your cover letter; we will change the online submission form on your behalf.

5. We note that your Data Availability Statement is currently as follows: “All relevant data are within the manuscript and its Supporting Information files.”

Please confirm at this time whether or not your submission contains all raw data required to replicate the results of your study. Authors must share the “minimal data set” for their submission. PLOS defines the minimal data set to consist of the data required to replicate all study findings reported in the article, as well as related metadata and methods (https://journals.plos.org/plosone/s/data-availability#loc-minimal-data-set-definition).

For example, authors should submit the following data:

- The values behind the means, standard deviations and other measures reported;

- The values used to build graphs;

- The points extracted from images for analysis.

Authors do not need to submit their entire data set if only a portion of the data was used in the reported study.

If your submission does not contain these data, please either upload them as Supporting Information files or deposit them to a stable, public repository and provide us with the relevant URLs, DOIs, or accession numbers. For a list of recommended repositories, please see https://journals.plos.org/plosone/s/recommended-repositories.

If there are ethical or legal restrictions on sharing a de-identified data set, please explain them in detail (e.g., data contain potentially sensitive information, data are owned by a third-party organization, etc.) and who has imposed them (e.g., an ethics committee). Please also provide contact information for a data access committee, ethics committee, or other institutional body to which data requests may be sent. If data are owned by a third party, please indicate how others may request data access.

6. One of the noted authors is a group or consortium “NOVELL Redesign”. In addition to naming the author group, please list the individual authors and affiliations within this group in the acknowledgments section of your manuscript. Please also indicate clearly a lead author for this group along with a contact email address.

7. Your ethics statement should only appear in the Methods section of your manuscript. If your ethics statement is written in any section besides the Methods, please move it to the Methods section and delete it from any other section. Please ensure that your ethics statement is included in your manuscript, as the ethics statement entered into the online submission form will not be published alongside your manuscript.

8. We note that Figures 2, 8, 9, 10 and 11 in your submission contain copyrighted images. All PLOS content is published under the Creative Commons Attribution License (CC BY 4.0), which means that the manuscript, images, and Supporting Information files will be freely available online, and any third party is permitted to access, download, copy, distribute, and use these materials in any way, even commercially, with proper attribution. For more information, see our copyright guidelines: http://journals.plos.org/plosone/s/licenses-and-copyright.

We require you to either (1) present written permission from the copyright holder to publish these figures specifically under the CC BY 4.0 license, or (2) remove the figures from your submission:

1. You may seek permission from the original copyright holder of Figure(s) [#] to publish the content specifically under the CC BY 4.0 license.

We recommend that you contact the original copyright holder with the Content Permission Form (http://journals.plos.org/plosone/s/file?id=7c09/content-permission-form.pdf) and the following text:

“I request permission for the open-access journal PLOS ONE to publish XXX under the Creative Commons Attribution License (CCAL) CC BY 4.0 (http://creativecommons.org/licenses/by/4.0/). Please be aware that this license allows unrestricted use and distribution, even commercially, by third parties. Please reply and provide explicit written permission to publish XXX under a CC BY license and complete the attached form.”

Please upload the completed Content Permission Form or other proof of granted permissions as an "Other" file with your submission.

In the figure caption of the copyrighted figure, please include the following text: “Reprinted from [ref] under a CC BY license, with permission from [name of publisher], original copyright [original copyright year].”

2. If you are unable to obtain permission from the original copyright holder to publish these figures under the CC BY 4.0 license or if the copyright holder’s requirements are incompatible with the CC BY 4.0 license, please either i) remove the figure or ii) supply a replacement figure that complies with the CC BY 4.0 license. Please check copyright information on all replacement figures and update the figure caption with source information. If applicable, please specify in the figure caption text when a figure is similar but not identical to the original image and is therefore for illustrative purposes only.

9. We note that Figures 3, 4, 5 and 10 includes an image of a [patient / participant / in the study].

As per the PLOS ONE policy (http://journals.plos.org/plosone/s/submission-guidelines#loc-human-subjects-research) on papers that include identifying, or potentially identifying, information, the individual(s) or parent(s)/guardian(s) must be informed of the terms of the PLOS open-access (CC-BY) license and provide specific permission for publication of these details under the terms of this license. Please download the Consent Form for Publication in a PLOS Journal (http://journals.plos.org/plosone/s/file?id=8ce6/plos-consent-form-english.pdf). The signed consent form should not be submitted with the manuscript, but should be securely filed in the individual's case notes. Please amend the methods section and ethics statement of the manuscript to explicitly state that the patient/participant has provided consent for publication: “The individual in this manuscript has given written informed consent (as outlined in PLOS consent form) to publish these case details”.

If you are unable to obtain consent from the subject of the photograph, you will need to remove the figure and any other textual identifying information or case descriptions for this individual.

10. We note that this data set consists of interview transcripts. Can you please confirm that all participants gave consent for interview transcript to be published?

If they DID provide consent for these transcripts to be published, please also confirm that the transcripts do not contain any potentially identifying information (or let us know if the participants consented to having their personal details published and made publicly available). We consider the following details to be identifying information:

- Names, nicknames, and initials

- Age more specific than round numbers

- GPS coordinates, physical addresses, IP addresses, email addresses

- Information in small sample sizes (e.g. 40 students from X class in X year at X university)

- Specific dates (e.g. visit dates, interview dates)

- ID numbers

Or, if the participants DID NOT provide consent for these transcripts to be published:

- Provide a de-identified version of the data or excerpts of interview responses

- Provide information regarding how these transcripts can be accessed by researchers who meet the criteria for access to confidential data, including:

a) the grounds for restriction

b) the name of the ethics committee, Institutional Review Board, or third-party organization that is imposing sharing restrictions on the data

c) a non-author, institutional point of contact that is able to field data access queries, in the interest of maintaining long-term data accessibility.

d) Any relevant data set names, URLs, DOIs, etc. that an independent researcher would need in order to request your minimal data set.

For further information on sharing data that contains sensitive participant information, please see: https://journals.plos.org/plosone/s/data-availability#loc-human-research-participant-data-and-other-sensitive-data

If there are ethical, legal, or third-party restrictions upon your dataset, you must provide all of the following details (https://journals.plos.org/plosone/s/data-availability#loc-acceptable-data-access-restrictions):

1. A complete description of the dataset

2. The nature of the restrictions upon the data (ethical, legal, or owned by a third party) and the reasoning behind them

3. The full name of the body imposing the restrictions upon your dataset (ethics committee, institution, data access committee, etc)

4. If the data are owned by a third party, confirmation of whether the authors received any special privileges in accessing the data that other researchers would not have

5. Direct, non-author contact information (preferably email) for the body imposing the restrictions upon the data, to which data access requests can be sent

11. If the reviewer comments include a recommendation to cite specific previously published works, please review and evaluate these publications to determine whether they are relevant and should be cited. There is no requirement to cite these works unless the editor has indicated otherwise.

[Note: HTML markup is below. Please do not edit.]

Reviewers' comments:

Reviewer's Responses to Questions

**Comments to the Author**

1. Is the manuscript technically sound, and do the data support the conclusions?

Reviewer #1: Yes

Reviewer #2: Partly

**********

2. Has the statistical analysis been performed appropriately and rigorously?

Reviewer #1: Yes

Reviewer #2: No

**********

3. Have the authors made all data underlying the findings in their manuscript fully available?

The PLOS Data policy

Reviewer #1: Yes

Reviewer #2: Yes

**********

4. Is the manuscript presented in an intelligible fashion and written in standard English?

Reviewer #1: Yes

Reviewer #2: Yes

**********

Reviewer #1: Thank you for your invitation to review the manuscript “Ending the Single versus Multi-Bedroom Debate: Hybrid Rooms Meet Stroke Rehabilitation Needs” for PLOS ONE.

With due respect, I believe the paper requires a revision before it can be considered for publication…

1. The paper, though conceptually interesting, lacks a clear hypothesis and defined research gap. The “hybrid room” concept is presented as an outcome rather than a solution derived from a well-structured problem statement. Authors should clarify the specific research question driving the study and how it advances beyond previous work on stroke-rehabilitation environments and evidence-based healthcare design???

2. While the “Provotype Esquissé Workshops” and VR simulations are detailed in narrative form, the analytical rigor is missing. The manuscript must specify the quantitative framework, e.g., …sample size justification, statistical validation of responses, reliability measures, and criteria used for performance comparison between room types.

3. The paper mentions 20 participants but gives no information on inclusion criteria, demographic distribution, prior VR experience, or potential bias introduced by training or familiarity with design software. The authors should demonstrate whether the sample size is statistically adequate for deriving meaningful conclusions.

4. Graphical data (e.g., Figure 12) are mentioned but not accompanied by statistical indicators such as confidence intervals, p-values, or effect sizes. Without these, the claimed superiority of the “Butterfly” or “Quartet” designs remains subjective.

5. The terminology “hybrid rooms” and “provotypes” needs rigorous definition and comparison with existing healthcare architectural typologies. It is unclear how these differ structurally or functionally from established design principles or prior adaptive modular systems used in stroke rehabilitation units.

6. The study would be strengthened by referencing or aligning findings with measurable rehabilitation indicators, mobility improvement, patient satisfaction, safety metrics, or recovery duration.

7. Statements about long-term cost efficiency, patient satisfaction, and improved outcomes are speculative without quantitative or longitudinal evidence. Authors should carefully delimit the claims to what their data can substantiate.

8. The literature review, though broad, does not sufficiently integrate recent systematic evidence from neuro-architecture and rehabilitation design studies.

9. Ethical approval and participant consent are briefly mentioned but lack details about data handling, anonymization, and reproducibility of the digital VR environments, which is crucial for research transparency under PLOS ONE standards.

10. Finally, the language and tone of the manuscript are sometimes promotional and design-studio-oriented rather than scientific…

Reviewer #2: Thank you for submitting your manuscript to this journal. This manuscript presents an innovative and interdisciplinary study addressing the longstanding single versus multi-bed debate in stroke rehabilitation environments. The integration of speculative design workshops, BIM modeling, and immersive VR testing represents a strong methodological contribution. The study is clearly written, logically structured, and grounded in prior work on value-focused design and rehabilitation architecture. However, several methodological and analytical concerns limit the strength of the conclusions in their current form. The following are my comments about this manuscript.

First, the study includes only 20 participants in the VR evaluation phase. While purposive sampling of experts and stroke survivors is reasonable for exploratory design research, the manuscript makes comparative claims regarding the superiority of specific room types. Without justification of sample adequacy or power considerations, these claims appear stronger than the data can support.

Second, the statistical analysis is limited to descriptive statistics. Median and interquartile range are reported, but no inferential tests were performed to examine differences between room types. Given that participants evaluated all four designs, a non-parametric repeated-measures analysis such as a Friedman test would have been appropriate. Post hoc comparisons could further clarify whether observed differences are statistically meaningful rather than impressionistic.

Third, the Visual Analogue Scale instrument appears to have been adapted for this study, yet no information is provided regarding reliability, internal consistency, or construct validity. The manuscript would benefit from reporting psychometric properties or at least acknowledging the exploratory nature of these measures.

Fourth, participant recruitment relied on existing collaborative networks. While understandable, this introduces potential bias, including familiarity bias and design-team proximity bias. The manuscript should discuss this more explicitly as a limitation.

Fifth, several claims in the discussion extend beyond the measured outcomes. The study evaluates perceived suitability and stakeholder preference within VR environments. It does not measure clinical recovery, healthcare cost reduction, or long-term functional outcomes. Statements suggesting improved recovery efficiency, reduced long-term disability, or cost savings should be reframed as hypotheses rather than conclusions.

**********

what does this mean?). If published, this will include your full peer review and any attached files.

If you choose “no”, your identity will remain anonymous but your review may still be made public.

**Do you want your identity to be public for this peer review?**  For information about this choice, including consent withdrawal, please see our Privacy Policy

Reviewer #1: No

Reviewer #2: No

**********

[NOTE: If reviewer comments were submitted as an attachment file, they will be attached to this email and accessible via the submission site. Please log into your account, locate the manuscript record, and check for the action link "View Attachments". If this link does not appear, there are no attachment files.]

To ensure your figures meet our technical requirements, please review our figure guidelines: https://journals.plos.org/plosone/s/figures

You may also use PLOS’s free figure tool, NAAS, to help you prepare publication quality figures: https://journals.plos.org/plosone/s/figures#loc-tools-for-figure-preparation.

NAAS will assess whether your figures meet our technical requirements by comparing each figure against our figure specifications.

# Author comment: Author response to Decision Letter 1

**DOI:** 10.1371/journal.pone.0356623.r002

4 May 2026

Our responses to the reviewers have been provided in the attached Response to Reviewers letter.

Attachment Submitted filename: Response to Reviewers.docx

# Peer review: Decision Letter 1

**DOI:** 10.1371/journal.pone.0356623.r003

**Authors:** Ruixin Liang

14 May 2026

Dear Dr. Lam,

Thank you for submitting your manuscript to PLOS ONE. After careful consideration, we feel that it has merit but does not fully meet PLOS ONE’s publication criteria as it currently stands. Therefore, we invite you to submit a revised version of the manuscript that addresses the points raised during the review process.

Please submit your revised manuscript by Jun 28 2026 11:59PM. If you will need more time than this to complete your revisions, please reply to this message or contact the journal office at plosone@plos.org. When you're ready to submit your revision, log on to https://www.editorialmanager.com/pone/ and select the 'Submissions Needing Revision' folder to locate your manuscript file.

- A letter that responds to each point raised by the academic editor and reviewer(s). You should upload this letter as a separate file labeled 'Response to Reviewers'.
- A marked-up copy of your manuscript that highlights changes made to the original version. You should upload this as a separate file labeled 'Revised Manuscript with Track Changes'.
- An unmarked version of your revised paper without tracked changes. You should upload this as a separate file labeled 'Manuscript'.

If you would like to make changes to your financial disclosure, please include your updated statement in your cover letter. Guidelines for resubmitting your figure files are available below the reviewer comments at the end of this letter.

If applicable, we recommend that you deposit your laboratory protocols in protocols.io to enhance the reproducibility of your results. Protocols.io assigns your protocol its own identifier (DOI) so that it can be cited independently in the future. For instructions see: https://journals.plos.org/plosone/s/submission-guidelines#loc-laboratory-protocols. Additionally, PLOS ONE offers an option for publishing peer-reviewed Lab Protocol articles, which describe protocols hosted on protocols.io. Read more information on sharing protocols at https://plos.org/protocols?utm_medium=editorial-email&utm_source=authorletters&utm_campaign=protocols.

As the corresponding author, your ORCID iD is verified in the submission system and will appear in the published article. PLOS supports the use of ORCID, and we encourage all coauthors to register for an ORCID iD and use it as well. Please encourage your coauthors to verify their ORCID iD within the submission system before final acceptance, as unverified ORCID iDs will not appear in the published article. *Only*  the individual author can complete the verification step; PLOS staff *cannot*  verify ORCID iDs on behalf of authors.

We look forward to receiving your revised manuscript.

Kind regards,

Ruixin Liang

Guest Editor

PLOS One

Journal Requirements:

If the reviewer comments include a recommendation to cite specific previously published works, please review and evaluate these publications to determine whether they are relevant and should be cited. There is no requirement to cite these works unless the editor has indicated otherwise.

Please review your reference list to ensure that it is complete and correct. If you have cited papers that have been retracted, please include the rationale for doing so in the manuscript text, or remove these references and replace them with relevant current references. Any changes to the reference list should be mentioned in the rebuttal letter that accompanies your revised manuscript. If you need to cite a retracted article, indicate the article’s retracted status in the References list and also include a citation and full reference for the retraction notice.

[Note: HTML markup is below. Please do not edit.]

Reviewers' comments:

Reviewer's Responses to Questions

**Comments to the Author**

Reviewer #1: All comments have been addressed

Reviewer #2: All comments have been addressed

**********

2. Is the manuscript technically sound, and do the data support the conclusions?

Reviewer #1: Yes

Reviewer #2: Yes

**********

3. Has the statistical analysis been performed appropriately and rigorously?

Reviewer #1: Yes

Reviewer #2: N/A

**********

4. Have the authors made all data underlying the findings in their manuscript fully available?

The PLOS Data policy

Reviewer #1: Yes

Reviewer #2: Yes

**********

5. Is the manuscript presented in an intelligible fashion and written in standard English?

Reviewer #1: Yes

Reviewer #2: Yes

**********

Reviewer #1: Revised version has been improved and is recommended for the publication. Great efforts by the authors.

Reviewer #2: Thank you for the comprehensive revision. The manuscript has improved substantially, and the methodological transparency is now much clearer. The study is technically sound within its stated exploratory and proof-of-concept scope. The justification of sample size, clarification of recruitment strategy, and more cautious framing of conclusions have strengthened the manuscript considerably.

The use of a values-based design brief, combined with immersive VR evaluation and multi-stakeholder input, represents a meaningful contribution to healthcare architecture and evidence-based design research. The data presented support the conclusions as framed, particularly because the study positions itself as exploratory and preference-based rather than confirmatory or outcome-driven. Specifically, I suggest:

1. Slightly tempering any remaining language in the abstract and conclusion that may imply direct improvement in clinical outcomes, clarifying that these findings indicate perceived suitability and stakeholder preference rather than measured rehabilitation effects.

2. Briefly expanding the discussion of potential bias due to prior participant involvement in the NOVELL project.

3. Adding one clarifying sentence regarding the robustness of the ranking aggregation method to enhance transparency.

These are minor clarifications rather than substantive changes.

**********

what does this mean?). If published, this will include your full peer review and any attached files.

If you choose “no”, your identity will remain anonymous but your review may still be made public.

**Do you want your identity to be public for this peer review?**  For information about this choice, including consent withdrawal, please see our Privacy Policy

Reviewer #1: No

Reviewer #2: No

**********

[NOTE: If reviewer comments were submitted as an attachment file, they will be attached to this email and accessible via the submission site. Please log into your account, locate the manuscript record, and check for the action link "View Attachments". If this link does not appear, there are no attachment files.]

To ensure your figures meet our technical requirements, please review our figure guidelines: https://journals.plos.org/plosone/s/figures

You may also use PLOS’s free figure tool, NAAS, to help you prepare publication quality figures: https://journals.plos.org/plosone/s/figures#loc-tools-for-figure-preparation.

NAAS will assess whether your figures meet our technical requirements by comparing each figure against our figure specifications.

# Author comment: Author response to Decision Letter 2

**DOI:** 10.1371/journal.pone.0356623.r004

22 May 2026

Our response to the reviewers questions as follows:

Reviewer #1: Revised version has been improved and is recommended for the publication. Great efforts by the authors.

Reviewer #2: Thank you for the comprehensive revision. The manuscript has improved substantially, and the methodological transparency is now much clearer. The study is technically sound within its stated exploratory and proof-of-concept scope. The justification of sample size, clarification of recruitment strategy, and more cautious framing of conclusions have strengthened the manuscript considerably.

The use of a values-based design brief, combined with immersive VR evaluation and multi-stakeholder input, represents a meaningful contribution to healthcare architecture and evidence-based design research. The data presented support the conclusions as framed, particularly because the study positions itself as exploratory and preference-based rather than confirmatory or outcome-driven. Specifically, I suggest:

1. Slightly tempering any remaining language in the abstract and conclusion that may imply direct improvement in clinical outcomes, clarifying that these findings indicate perceived suitability and stakeholder preference rather than measured rehabilitation effects.

Agree: We have edited the abstract and conclusions accordingly.

The abstract (p2) now reads:

Stroke is a leading cause of disability worldwide, and stroke rehabilitation plays a critical role in recovery. Early rehabilitation often occurs in a hospital setting, but the hospital built environment is rarely designed to support the specific needs and priorities of people who have had a stroke. In recent decades, there has been an ongoing debate regarding the benefits of single versus multi-bed rooms in healthcare settings. This debate centres around balancing privacy and rest, with social interaction and safety. While single rooms may be beneficial for some people, multi-bed rooms may be better for others. Both options, however, come with their own set of drawbacks in the context of stroke rehabilitation.

In this study, we conducted a collaborative Living Lab to design a series of provocative-prototype hybrid room typologies that merged different aspects of single and multi-bed rooms to provide options specific for stroke rehabilitation. These typologies were used to generate four alternative bedroom designs which were modelled in virtual reality. The designs were informed by a values-based brief, outlining a set of predefined key criteria for optimising stroke rehabilitation environments: opportunities for activity and rest, wellbeing, safety, efficiency, flexibility, personal control, access to outdoors and green space, and access to a positive and stimulating environment. Twenty lived-experience and professional experts evaluated these virtual designs against the predefined values-based criteria.

Evaluation revealed two preferred hybrid hospital room designs that incorporated beneficial features of both multi-bed and single-bed rooms. Our approach of values-based design and evaluation within a virtual environment proved an effective means of generating and evaluating design options with meaningful input from a wide range of stakeholders. These findings highlight the potential for flexible and adaptable room design to meet the specific needs and priorities of people participating in early-stage rehabilitation after stroke.

The Conclusion (p24) now reads:

Our findings demonstrate the potential for using hybrid rooms to achieve the best of both Single and Multi-Bedroom types in a hospital setting. To address the Single vs. Multi-Bedroom debate in stroke rehabilitation, we co-designed and evaluated hypothetical hybrid room designs aimed at meeting objectives identified by stakeholders. We integrated input from multiple stakeholders, combining lived experiences, operational insights, and functional ideas to design hybrid rooms that merge the advantages of both single- and multi-bed rooms. Technologies like BIM, VR, and game engines allowed stakeholders to view and evaluate the designs.

Our findings show the importance of flexibility in bedroom designs; hybrid rooms allow people who have had a stroke to adjust privacy and social interaction levels, aligning with the dynamic needs of stroke rehabilitation. Space and cost trade-offs are also important considerations. While hybrid designs like the Butterfly and Quartet require more spatial and initial financial investment than standard rooms, their perceived appropriateness for the stroke rehabilitation context may justify the expenditure.

The future of stroke rehabilitation room design should move beyond the single versus multi-bed debate and instead focus on adaptable, patient-centered solutions that balance privacy, social interaction, safety, and therapeutic functionality. Further research should explore the long-term clinical and economic impacts of hybrid rooms to inform evidence-based design guidelines. The focus of hospital bedroom design and any design evaluation should not be on the number of beds in the room, but rather, on whether the room meets the specific clinical and wellbeing needs of the users.

2. Briefly expanding the discussion of potential bias due to prior participant involvement in the NOVELL project.

Agree: We have elaborated on this further in the Limitations (pg. 23). In addition, to check for familiarity bias, we have calculated the aggregated rankings for two subgroups: 1) participants who had participated in previous NOVELL workshops (non-novices) and 2) participants who were novice to the project. Reassuringly, we have found that the butterfly and quartet designs have remained in the top two rankings, and the duet has the lowest ranking, for all the predefined objectives for both of these subgroups. We have added this to the Methods (pg. 11), Results (pg. 18) and Limitations (pg. 23). This section of the Limitations now reads:

While we included a mix of participants familiar with the project and those who were unfamiliar with the project (novices), it should be noted that participant recruitment relied on existing collaborative networks. None of the participants who were familiar with the NOVELL project (non-novices) had seen the final provotypes prior to evaluating them in VR, but some were involved in the initial speculative design esquisses. The network-based recruitment and involvement in earlier stages of the project introduces potential bias, including research-team proximity and familiarity bias which may skew results in favor of designs or concepts that participants were already familiar with. Participants did not, however, favor the duet design, despite this design arguably being the most familiar as it drew heavily from currently Australian guidelines and norms. Reassuringly, we did not observe any notable differences in responses between participants who were familiar with the project (non-novices) versus those who were unfamiliar (novices); aggregated rankings of the bedroom provotypes for both subgroups placed the butterfly and quartet provotypes in the top two. In future expansion of this research, however, wider reaching recruitment methods should be included to ensure a wide range of opinions, including people who are not familiar with the priorities and objectives that were defined in the NOVELL project.

3. Adding one clarifying sentence regarding the robustness of the ranking aggregation method to enhance transparency.

Agree: We thank the reviewer for this recommendation. A sentence has been added to the Methods (pg. 11) to describe the robustness of the aggregation algorithm compared to other methods for combining rankings: “This aggregation method is a robust way of combining participant’s rankings because it treats rankings as purely ordinal data and uses graph theory to extract only the ordering that the data genuinely support—i.e., allowing ties between the provotypes—rather than summing ranks or forcing a definitive ranking [42].”

These are minor clarifications rather than substantive changes.

Attachment Submitted filename: Response_to_reviewers_auresp_2.docx

# Peer review: Decision Letter 2

**DOI:** 10.1371/journal.pone.0356623.r005

**Authors:** Ruixin Liang

27 May 2026

Thank you for submitting your manuscript to PLOS ONE. After careful consideration, we feel that it has merit but does not fully meet PLOS ONE’s publication criteria as it currently stands. Therefore, we invite you to submit a revised version of the manuscript that addresses the points raised during the review process.

Please submit your revised manuscript by Jul 11 2026 11:59PM. If you will need more time than this to complete your revisions, please reply to this message or contact the journal office at plosone@plos.org. When you're ready to submit your revision, log on to https://www.editorialmanager.com/pone/ and select the 'Submissions Needing Revision' folder to locate your manuscript file.

- A letter that responds to each point raised by the academic editor and reviewer(s). You should upload this letter as a separate file labeled 'Response to Reviewers'.
- A marked-up copy of your manuscript that highlights changes made to the original version. You should upload this as a separate file labeled 'Revised Manuscript with Track Changes'.
- An unmarked version of your revised paper without tracked changes. You should upload this as a separate file labeled 'Manuscript'.

If you would like to make changes to your financial disclosure, please include your updated statement in your cover letter. Guidelines for resubmitting your figure files are available below the reviewer comments at the end of this letter.

If applicable, we recommend that you deposit your laboratory protocols in protocols.io to enhance the reproducibility of your results. Protocols.io assigns your protocol its own identifier (DOI) so that it can be cited independently in the future. For instructions see: https://journals.plos.org/plosone/s/submission-guidelines#loc-laboratory-protocols. Additionally, PLOS ONE offers an option for publishing peer-reviewed Lab Protocol articles, which describe protocols hosted on protocols.io. Read more information on sharing protocols at https://plos.org/protocols?utm_medium=editorial-email&utm_source=authorletters&utm_campaign=protocols.

As the corresponding author, your ORCID iD is verified in the submission system and will appear in the published article. PLOS supports the use of ORCID, and we encourage all coauthors to register for an ORCID iD and use it as well. Please encourage your coauthors to verify their ORCID iD within the submission system before final acceptance, as unverified ORCID iDs will not appear in the published article. *Only*  the individual author can complete the verification step; PLOS staff *cannot*  verify ORCID iDs on behalf of authors.

We look forward to receiving your revised manuscript.

Kind regards,

Ruixin Liang

Guest Editor

PLOS One

Journal Requirements:

1. If the reviewer comments include a recommendation to cite specific previously published works, please review and evaluate these publications to determine whether they are relevant and should be cited. There is no requirement to cite these works unless the editor has indicated otherwise.

2. Please review your reference list to ensure that it is complete and correct. If you have cited papers that have been retracted, please include the rationale for doing so in the manuscript text, or remove these references and replace them with relevant current references. Any changes to the reference list should be mentioned in the rebuttal letter that accompanies your revised manuscript. If you need to cite a retracted article, indicate the article’s retracted status in the References list and also include a citation and full reference for the retraction notice.

[Note: HTML markup is below. Please do not edit.]

Reviewer's Responses to Questions

**Comments to the Author**

Reviewer #2: All comments have been addressed

**********

2. Is the manuscript technically sound, and do the data support the conclusions?

Reviewer #2: Yes

**********

3. Has the statistical analysis been performed appropriately and rigorously?

Reviewer #2: N/A

**********

4. Have the authors made all data underlying the findings in their manuscript fully available?

The PLOS Data policy

Reviewer #2: Yes

**********

5. Is the manuscript presented in an intelligible fashion and written in standard English?

Reviewer #2: Yes

**********

Reviewer #2: Thank you for the careful and comprehensive revision. The manuscript has improved substantially across multiple areas, particularly in relation to methodological transparency, interpretation of findings, and acknowledgement of study limitations. I suggest reviewing a few remaining statements in the Highlights section and selected discussion wording that may still slightly imply direct therapeutic or clinical superiority rather than perceived suitability and stakeholder preference.

**********

what does this mean?). If published, this will include your full peer review and any attached files.

If you choose “no”, your identity will remain anonymous but your review may still be made public.

**Do you want your identity to be public for this peer review?**  For information about this choice, including consent withdrawal, please see our Privacy Policy

Reviewer #2: No

**********

[NOTE: If reviewer comments were submitted as an attachment file, they will be attached to this email and accessible via the submission site. Please log into your account, locate the manuscript record, and check for the action link "View Attachments". If this link does not appear, there are no attachment files.]

To ensure your figures meet our technical requirements, please review our figure guidelines: https://journals.plos.org/plosone/s/figures

You may also use PLOS’s free figure tool, NAAS, to help you prepare publication quality figures: https://journals.plos.org/plosone/s/figures#loc-tools-for-figure-preparation.

NAAS will assess whether your figures meet our technical requirements by comparing each figure against our figure specifications.

# Author comment: Author response to Decision Letter 3

**DOI:** 10.1371/journal.pone.0356623.r006

2 Jun 2026

In response to the Reviewer's comments, we offer the following response:

Reviewer #2: Thank you for the careful and comprehensive revision. The manuscript has improved substantially across multiple areas, particularly in relation to methodological transparency, interpretation of findings, and acknowledgement of study limitations. I suggest reviewing a few remaining statements in the Highlights section and selected discussion wording that may still slightly imply direct therapeutic or clinical superiority rather than perceived suitability and stakeholder preference.

Agree: We thank the reviewer for highlighting this to us. We have removed the ‘Highlights’ section as it is not a requirement of PlosOne and we have covered these points within the contents of the paper. We have updated the discussion section with additional clarifications and changing or removing any wording that may have slightly implied direct therapeutic or clinical superiority rather than perceived suitability and stakeholder preference.

Attachment Submitted filename: Response to reviewers.pdf

# Peer review: Decision Letter 3

**DOI:** 10.1371/journal.pone.0356623.r007

**Authors:** Ruixin Liang

5 Aug 2026

Ending the single versus multi-bedroom debate: hybrid rooms meet stroke rehabilitation needs

PONE-D-25-63722R3

Dear Dr. Lam,

We’re pleased to inform you that your manuscript has been judged scientifically suitable for publication and will be formally accepted for publication once it meets all outstanding technical requirements.

Within one week, you’ll receive an e-mail detailing the required amendments. When these have been addressed, you’ll receive a formal acceptance letter and your manuscript will be scheduled for publication.

An invoice will be generated when your article is formally accepted. Please note, if your institution has a publishing partnership with PLOS and your article meets the relevant criteria, all or part of your publication costs will be covered. Please make sure your user information is up-to-date by logging into Editorial Manager at Editorial Manager® and clicking the ‘Update My Information' link at the top of the page. For questions related to billing, please contact billing support.

If your institution or institutions have a press office, please notify them about your upcoming paper to help maximize its impact. If they’ll be preparing press materials, please inform our press team as soon as possible -- no later than 48 hours after receiving the formal acceptance. Your manuscript will remain under strict press embargo until 2 pm Eastern Time on the date of publication. For more information, please contact onepress@plos.org.

Kind regards,

Ruixin Liang

Guest Editor

PLOS One

Additional Editor Comments (optional):

Reviewers' comments:

Reviewer's Responses to Questions

**Comments to the Author**

Reviewer #2: All comments have been addressed

**********

2. Is the manuscript technically sound, and do the data support the conclusions?

Reviewer #2: Yes

**********

3. Has the statistical analysis been performed appropriately and rigorously?

Reviewer #2: N/A

**********

4. Have the authors made all data underlying the findings in their manuscript fully available?

The PLOS Data policy

Reviewer #2: Yes

**********

5. Is the manuscript presented in an intelligible fashion and written in standard English?

Reviewer #2: Yes

**********

Reviewer #2: Thank you for the comprehensive revision and detailed responses to reviewer comments. The manuscript has improved substantially across multiple review rounds. The authors have adequately addressed concerns regarding methodological transparency, interpretation of findings, potential familiarity bias, and the framing of conclusions. The removal of wording that could imply direct therapeutic superiority has strengthened the manuscript and ensured that the conclusions remain appropriately aligned with the exploratory nature of the study. I have no further substantive concerns and believe the manuscript is suitable for publication in its current form.

**********

what does this mean?). If published, this will include your full peer review and any attached files.

If you choose “no”, your identity will remain anonymous but your review may still be made public.

**Do you want your identity to be public for this peer review?**  For information about this choice, including consent withdrawal, please see our Privacy Policy

Reviewer #2: No

**********

<table border="0" cellpadding="0" cellspacing="0" class="datatable3" style="border-collapse: collapse; width: 678px; line-height: 14px; color: rgb(0, 0, 51); font-family: verdana, geneva, arial, helvetica, sans-serif; font-size: 11.2px;"><tbody><tr style="background-color: rgb(244, 244, 244);"><td style="padding: 3px; border: 1px solid rgb(255, 255, 255);">Mark Huen Choong Huen Choong Lam</td></tr></tbody></table>

# Editor report: Acceptance letter

**DOI:** 10.1371/journal.pone.0356623.r008

**Authors:** Ruixin Liang

PONE-D-25-63722R3

PLOS One

Dear Dr. Lam,

I'm pleased to inform you that your manuscript has been deemed suitable for publication in PLOS One. Congratulations! Your manuscript is now being handed over to our production team.

At this stage, our production department will prepare your paper for publication. This includes ensuring the following:

* All references, tables, and figures are properly cited

* All relevant supporting information is included in the manuscript submission,

* There are no issues that prevent the paper from being properly typeset

You will receive further instructions from the production team, including instructions on how to review your proof when it is ready. Please keep in mind that we are working through a large volume of accepted articles, so please give us a few days to review your paper and let you know the next and final steps.

Lastly, if your institution or institutions have a press office, please let them know about your upcoming paper now to help maximize its impact. If they'll be preparing press materials, please inform our press team within the next 48 hours. Your manuscript will remain under strict press embargo until 2 pm Eastern Time on the date of publication. For more information, please contact onepress@plos.org.

You will receive an invoice from PLOS for your publication fee after your manuscript has reached the completed accept phase. If you receive an email requesting payment before acceptance or for any other service, this may be a phishing scheme. Learn how to identify phishing emails and protect your accounts at https://explore.plos.org/phishing.

If we can help with anything else, please email us at customercare@plos.org.

Thank you for submitting your work to PLOS One and supporting open access.

Kind regards,

PLOS One Editorial Office Staff

on behalf of

Dr. Ruixin Liang

Guest Editor

PLOS One
